# Optimal feedback control successfully explains changes in neural modulations during experiments with brain-machine interfaces

**DOI:** 10.3389/fnsys.2015.00071

**Published:** 2015-05-19

**Authors:** Miri Benyamini, Miriam Zacksenhouse

**Affiliations:** Brain-computer Interfaces for Rehabilitation Laboratory, Department of Mechanical Engineering, Technion - Israel Institute of TechnologyHaifa, Israel

**Keywords:** brain-machine interfaces, neural modulations, optimal feedback control, computational motor control, process noise

## Abstract

Recent experiments with brain-machine-interfaces (BMIs) indicate that the extent of neural modulations increased abruptly upon starting to operate the interface, and especially after the monkey stopped moving its hand. In contrast, neural modulations that are correlated with the kinematics of the movement remained relatively unchanged. Here we demonstrate that similar changes are produced by simulated neurons that encode the relevant signals generated by an optimal feedback controller during simulated BMI experiments. The optimal feedback controller relies on state estimation that integrates both visual and proprioceptive feedback with prior estimations from an internal model. The processing required for optimal state estimation and control were conducted in the state-space, and neural recording was simulated by modeling two populations of neurons that encode either only the estimated state or also the control signal. Spike counts were generated as realizations of doubly stochastic Poisson processes with linear tuning curves. The model successfully reconstructs the main features of the kinematics and neural activity during regular reaching movements. Most importantly, the activity of the simulated neurons successfully reproduces the observed changes in neural modulations upon switching to brain control. Further theoretical analysis and simulations indicate that increasing the process noise during normal reaching movement results in similar changes in neural modulations. Thus, we conclude that the observed changes in neural modulations during BMI experiments can be attributed to increasing process noise associated with the imperfect BMI filter, and, more directly, to the resulting increase in the variance of the encoded signals associated with state estimation and the required control signal.

## 1. Introduction

Brain-Machine Interfaces (BMIs) have been developed to provide a direct communication link between the brain and external devices, bypassing the remaining, potentially injured neuro-muscular system (Nicolelis, 2001; Taylor et al., 2002; Lebedev et al., 2005). Additionally, BMIs provide a unique window into information representation and processing in the brain. In particular, it was observed that the extent of neural modulations (during BMI experiments reported in Carmena et al., 2003) increased abruptly upon starting to operate the interface, and especially after the monkey stopped moving its hand (Zacksenhouse et al., 2007). In contrast, neural modulations that are correlated with the movement kinematics remained relatively unchanged. Here we develop an optimal feedback control model (OFC) of BMI experiments to explain the observed changes in neural modulations and to investigate how they are related to changes in state estimation during brain control.

OFC was recently proposed as a viable model for motor control during reaching movements (Todorov and Jordan, 2002; Todorov, 2005; Shadmehr and Krakauer, 2008). While the term “optimal feedback control” emphasizes the optimality of the control gains given the cost function, the main component of interest here is optimal state estimation. This component is hypothesized to integrate visual and proprioceptive information with prior state estimation from an internal model to optimize the posterior state estimate (Miall and Wolpert, 1996; Wolpert and Ghahramani, 2000). The relative weights given to the sensory measurements versus the internal estimation depend on the relative variance of measurement and process noise, and, for linear systems corrupted by Gaussian noise, are determined by the Kalman filter (Schwartz, 2004; Stengel, 2012). The main hypothesis of this paper is that changes in process and measurement noise caused by the switch to brain control can explain the observed changes in neural modulations.

OFC is adopted here, instead of alternative computational motor control models, such as feedback error learning (Kawato et al., 1987), active inference (Friston et al., 2010, 2011) and distal teacher (Jordan and Rumelhart, 1992), for three main reasons. First, OFC does not require explicit specification of the desired trajectory of movement. While the desired trajectory can be assumed to follow the minimum jerk profile, its specification during brain control, when the initial stroke does not reach the target, is not straightforward. In contrast, OFC generates the trajectory implicitly, rather than following an externally specified trajectory. Secondly, the optimization inherent in OFC constrains the parameters of the state estimation filter and the control gains, so the model has fewer free parameters that need to be tuned. Thus, OFC provides a coherent and principled framework for investigating possible mechanisms underlying changes in neural modulations following the transition to brain control. Finally, OFC explicitly accounts for the variance of the process and measurement noise, which are assumed to change when switching to brain control, and thus is most appropriated for investigating how these mechanisms may contribute to the observed changes in neural modulations.

The processing required for optimal state estimation and control are conducted in the state-space. Neural recording is simulated by modeling two populations of neurons that encode the relevant signals, including either just the estimated state or also the resulting control signal. Thus, the approach presented here combines the dynamical perspective, which focuses on how the brain commands movements, with the representational perspective, which investigates what the neurons encode (Shenoy et al., 2013), by suggesting that they encode the signals that are relevant for the computations that underlie state estimation and control. The goal of this work is to investigate whether the resulting neural activity would produce the observed changes in neural modulations during BMI experiments.

The above OFC framework for investigating the changes in neural modulations during BMI experiments should be distinguished from recent applications of state estimation (Schwartz, 2004; Wu et al., 2006; Cunningham et al., 2011) and OFC (Shanechi et al., 2013a,b) to improve neural decoders for BMIs. BMI decoders estimate the state by integrating the observed neural activity, which is assumed to encode the state of the movement, with a presumed or learned model of reaching movement dynamics. Advanced models (Shanechi et al., 2013a,b) account for the effect of the control signal generated by the brain on the movement dynamics by modeling the sensory motor system in the brain as OFC. However, since the focus of such models is the BMI decoder, the brain is assumed to know the actual state of the movement via noise-free sensory measurements, thereby eliminating the need for state estimation. In contrast, our focus is on state estimation in the brain, which is assumed to integrate noisy visual and proprioception observations with an internal model of movement dynamics (Miall and Wolpert, 1996; Wolpert and Ghahramani, 2000), and how the changes in sensory and process noise affect those estimations and the resulting neural activity.

**Figure 1:**
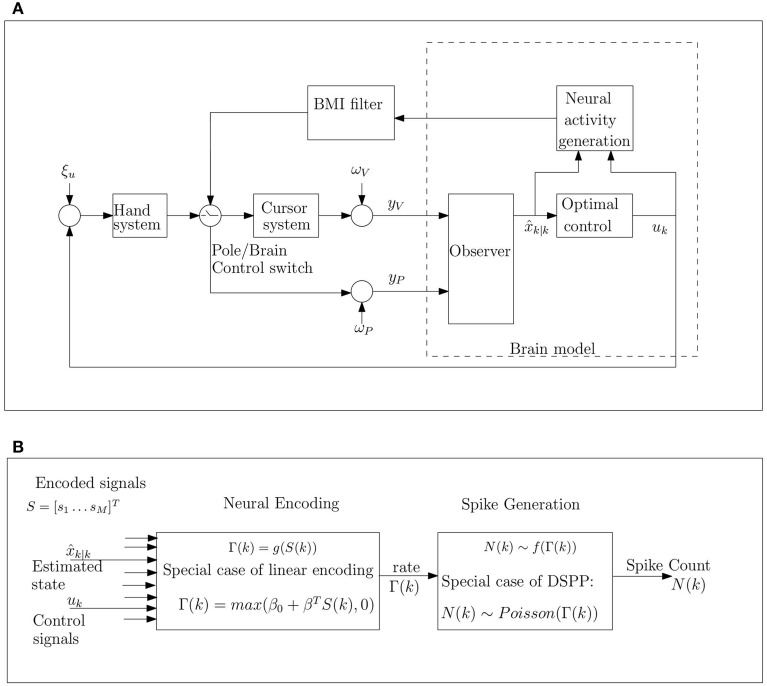
**Schematic model of movement control during BMI experiments under the hypothesis that the brain implements OFC (A), and detailed block diagram of neural activity generation (B)**. **(A)** The brain model receives noisy proprioceptive (*y*_*P*_) and visual (*y*_*C*_) measurements from the hand and cursor, corrupted by proprioceptive and visual measurement noise, ω_*P*_ and ω_*V*_, respectively. These noisy measurements are integrated with prior predictions from the internal model to generate optimal state estimates $\hat{x}$_*k*|*k*_ and control signal *u*_*k*_, which are encoded by the neural activity. The control signal is corrupted by hand process noise ξ_*u*_. The BMI filter is trained based on the neural activity in pole control and then used to move the cursor in brain control. **(B)** The cumulative bin rate, Γ(*k*), at time step *k*, is modulated by the encoded signals $S={\left[s_{1},\;\cdots \;,s_{M}\right]}^{T}$ including the estimated state $\hat{x}$_*k*|*k*_ and control signals *u*_*k*_. The spike-count *N*(*k*) is generated as a doubly stochastic Point process given the rate parameter Γ(*k*). Here we consider the special case of linear encoding, where Γ(*k*) is a linear combination of the encoded signals (including the estimated speed and the magnitude of the control signal), and doubly stochastic Poisson processes (DSPP), where the spike count *N*(*k*) has a Poisson distribution with rate Γ(*k*).

In summary, this work investigates the hypothesis that the observed changes in neural modulations following the transition to brain control can be explained in the context of OFC model of motor control. We hypothesize that neurons in cortical motor areas, and in particular in primary motor area, M1, and premotor dorsal, PMd, encode the relevant signals for OFC of reaching movements, i.e., the estimated state and the resulting control signal, as depicted in Figure 1. Thus, the observed changes in neural rate modulations are hypothesized to reflect corresponding changes in the variance of these signals. Furthermore, we hypothesize that the increase in the variance of the estimated state and control signal is due to the higher process noise during brain control caused by the imperfect BMI filter. These hypotheses are investigated in three levels: (i) simulations of pole control and brain control, and evaluation of the corresponding changes in neural modulations (Section 3.2), (ii) theoretical analysis of the effect of increasing process noise during pole control (Section 2.4), and (iii) simulations of the effect of increasing process noise on neural modulations during pole control (Section 3.3). Other aspects of the model are investigated in Sections 3.1, 3.4–3.6.

## 2. Materials and methods

### 2.1. Experimental methods

The proposed model is evaluated by comparing the different properties of the simulated neural activity to those observed during the BMI experiments described in Carmena et al. (2003). The BMI experiments were conducted with macaque monkeys whose goal was to move the cursor to randomly appearing targets and hold the cursor on the target for 150 ms to accept a juice reward. Each experiment included three control modes: (i) pole control during which the monkey controlled the cursor using hand-held pole, (ii) brain control with hand movements (BCWHM), during which the cursor was controlled by the output of the BMI interface while the monkey continued moving the pole and (iii) brain control without hand movements (BCWOHM), during which the cursor was controlled by the BMI interface even though the monkey stopped moving the pole.

Neural activity was recorded from multiple brain area, but mostly from the primary motor area (M1) and the dorsal premotor area (PMd). The BMI interface binned the recorded spike trains in 100 ms bins, to generate the input to a linear filter. The linear BMI filter was trained with data recorded during the last 10 min of pole control, and held fixed during brain control.

### 2.2. Analysis methods

#### 2.2.1. Percent overall modulations

Spike-trains can be considered as realizations of point processes (Johnson, 1996). The number of spikes recorded in a bin, *N*, depends on the cumulative spike-rate during the bin, Γ (Zacksenhouse et al., 2007), which can be modulated by the encoded signals as outlined in Figure 1B. Since this dependence is stochastic, the variance of the spike-count *var*(*N*) is higher than the variance of the cumulative spike-rate *var*(Γ) (Zacksenhouse et al., 2007). While the variance of the spike-count can be measured directly, it is the variance of the cumulative spike-rate that captures the effect of the encoded signals on rate modulations. In order to quantify these rate modulations, the percent overall modulation (POM) is defined as Zacksenhouse et al. (2007)

(1) $$POM=\frac{var[\Gamma ]}{var[N]}\cdot 100\%$$

Since the variance of the bin-rate cannot be measured directly, the POM cannot be estimated without further assumptions. Consider first the simplest point process that can describe stochastic rate modulations, i.e., the doubly stochastic Poisson processes (DSPP), for which Snyder (1975), Zacksenhouse et al. (2007)

(2) $$\begin{array}{l} \;E(N_{DSPP})=E(\Gamma )\\ Var(N_{DSPP})=var(\Gamma )+E(\Gamma ) \end{array}$$

In this case, the POM can be estimated form the mean and variance of the spike count as

(3) $$P\hat{O}M[N]=\frac{var[N]-E[N]}{var[N]}\;\cdot \;100\%$$

where $P\hat{O}M$ denotes the estimated POM. When applied to the analysis of spike trains recorded during BMI experiments, we assume that the movement is composed of an asynchronous sequence of reaching movements (Zacksenhouse et al., 2014) so the cumulative spike-rate and the binned spike-counts are stationary processes. Hence, the POM is estimated from Equation (3) using the temporal mean and variance rather than ensemble mean and variance.

In the general case of doubly stochastic Point processes (not necessarily Poisson distributed), the variance of the spike-counts can be decomposed into Churchland et al. (2011)

(4) var(N)=var[E[N|Γ]]+E[var[N|Γ]]

The first term on the right reflects the variance of the cumulative spike-rate Γ, though is equal to it only for DSPPs. The second term can be interpreted as the Point process noise, since it contributes to the variance of the spike-counts even if Γ is constant. While this term equals the mean rate only for DSPPs, evidence suggests that in many cases it is proportional to the mean rate (Tolhurst et al., 1983; Geisler and Albrecht, 1995), i.e., *var*[*N*|Γ] = γ *E*[*N*|Γ], (γ > 0) and hence *E*[*var*[*N*|Γ]] = γ *E*[*N*]. Hence, a revised definition of POM quantifies the ratio between the first term on the right, which reflects the variance of the cumulative spike-rate Γ, and the total variance in the spike counts

(5) $$POM_{REV}(N)=\frac{var[E[N|\Gamma ]]}{var(N)}\cdot 100\%$$

This can be related to the POM estimated by Equation (3) by inserting the expression for the point process noise in Equation (4) and Equation (5) to get

(6) $$P\hat{O}M_{REV}(N)\;=\;\frac{var[N]-\gamma E[N]}{var(N)}\;\cdot \;100\%\;=\;(\gamma P\hat{O}M-(1-\gamma ))\;\cdot \;100\%$$

Thus, changes in the POM estimated by Equation (3) reflect proportional changes in the revised POM of Equation (5) up to a positive scaling and positive or negative offset. Having established this connection, the revised POM will not be used any further.

All the POM results described in Section 3 and shown in the Figures are $P\hat{O}M$ (Equation 3), estimated from temporal statistics, while the theoretical analysis in Section 2.4 is based on the definition of POM in Equation (1).

#### 2.2.2. Percent kinematics modulations

Numerous studies suggested that the neural activity can be related to the kinematics of the movement, including position, velocity and speed, via a linear model (Georgopoulos et al., 1986; Ashe and Georgopoulos, 1994; Moran and Schwartz, 1999; Todorov, 2000; Zacksenhouse and Nemets, 2008; Chang et al., 2014)

(7) $$\begin{array}{l} N(k)=\sum_{l\;=\;-L_{1}}^{L_{2}}\omega_{p_{cx}}(l)p_{cx}(k+l)+\sum_{l\;=\;-L_{1}}^{L_{2}}\omega_{p_{cy}}(l)p_{cy}(k+l)\\ \;+\sum_{l\;=\;-L_{1}}^{L_{2}}\omega_{v_{cx}}(l)v_{cx}(k+l)+\sum_{l\;=\;-L_{1}}^{L_{2}}\omega_{v_{cy}}(l)v_{cy}(k+l)\\ \;+\sum_{l\;=\;-L_{1}}^{L_{2}}\omega_{S}(l)S_{pc}(k+l)+\omega_{0}+\epsilon (k) \end{array}$$

where *k* is the index of the current bin, *N*(*k*) is the binned spike count, *p*_*cx*_ and *p*_*cy*_ are the *x* and *y* components of the cursor position, *v*_*cx*_ and *v*_*cy*_ are the *x* and *y* components of the cursor velocity, $S_{pc}=\sqrt[{2}]{v_{cx}^{2}+v_{cy}^{2}}$ is the cursor's speed, *l* is the relative lag, *L*_1_ and *L*_2_ are the number of preceding and succeeding lags, ω_*p*_*cx*__, ω_*p*_*cy*__, ω_*v*_*cx*__, ω_*v*_*cy*__ and ω_*S*_ are the corresponding regression weights, ω_0_ is the bias parameters and ϵ(*k*) is the residual error.

The coefficient of determination of the spatio-temporal regression, *R*^2^, describes the fraction of the variance in the binned spike-count that is linearly related to variations in the temporal profile of the kinematic signals in the surrounding temporal window. Expressed as a percentage, *R*^2^ is referred to as the percent kinematic-related modulation, or PKM (Zacksenhouse et al., 2007). The PKM results reported here are computed with L1 = L2 = 9.

### 2.3. Modeling methods

The simplifying assumptions on which the proposed OFC model for BMI experiments is based are explicitly stated in Section 2.3.1. As depicted in Figure 1A, the model includes three main parts: (1) Hand and cursor model, (2) Brain model, and (3) BMI filter, as briefly explained in Sections 2.3.2–2.3.5, and detailed in Appendices A and B. Model parameters are detailed in Section 2.3.6, and the effect of process noise in pole control is investigated in Section 2.3.7.

#### 2.3.1. Simplifying assumptions

The goal of this work is to construct a simple model that can capture the observed abrupt changes in neural modulations following the transition to brain control, and to assess if those changes can be attributed to increasing process and measurement noise. Hence, within the framework of OFC (Todorov and Jordan, 2002; Todorov, 2005; Shadmehr and Krakauer, 2008) we made the following simplifying assumptions (SA):

**Table:** 

SA1:	Absolute delays are ignored, thought the relative time-shift between the command to the muscle and the force it produces is captured by a second order muscle model (Section 2.3.2). Thus, the model successfully reproduces the observed time-lag in the cross-correlation between the neural activity and the movement velocity (Lebedev et al., 2005) as detailed in Section 3.1. The effect of sensory delays on movement variability and bias can be accounted for by sensory noise, at least when the perturbations are not abrupt (Todorov and Jordan, 2002; Crevecoeur and Scott, 2013).
SA2:	The process noise during pole control is assumed to be signal independent white Gaussian noise. This assumption is justified since the resulting velocity profile agrees well with the commonly observed minimum jerk velocity profile (Flash and Hogan, 1985). While evidence suggests that the variance of the process noise increases with the control signal (Harris and Wolpert, 1998; Wolpert and Ghahramani, 2000), this was not modeled in order to focus on the effect of brain control on process noise and neural modulations.
SA3:	The internal forward model is assumed to be identical to the actual hand and cursor system in pole control. Adaptation to brain control is ignored since the focus is on the abrupt changes in neural modulations immediately after the transition to brain control. Adaptation is expected to be important in explaining the subsequent gradual decrease in neural modulations with BMI sessions (Zacksenhouse et al., 2007), and will be explored in future work.
SA4:	Spike counts are generated as realization of doubly stochastic Poisson process with linear tuning curves (as detailed in Section 2.3.4) (Zacksenhouse and Nemets, 2008; Cunningham et al., 2011; Chang et al., 2014).

#### 2.3.2. Simplified hand and cursor model

Following Todorov (2005) the hand is modeled as a point mass driven by an over damped second order muscle model that responds to the control signal from the brain. An additional friction term is introduced to model the friction of the hand held pole, as described by Equation (A2) in Supplementary Material.

As in the BMI experiments (Carmena et al., 2003), the cursor position during brain control is generated by integrating the velocity predicted by the BMI interface and filtering it with a high pass filter (HPF) to remove low frequency drifts, as described by Equation (A7) in Supplementary Material. During pole control, the cursor position is determined in the same way, using the actual hand velocity instead of the predicted velocity.

The simulation is updated at 100 Hz, and the discrete dynamics of the combined system, including the hand, cursor and target along a single degree of freedom, can be expressed as (see Equation A10 in Supplementary Material)

(8) $$x(k+1)=Ax(k)+B_{u}u(k)+B_{BMI}v_{BMI}(k)+\xi_{p}(k)$$

where *x* is the combined state (specified by Equation A9 in Supplementary Material), *A*, *B*_*u*_, and *B*_*BMI*_ are the matrices describing the system dynamics, the effect of the control signal *u* generated by the optimal feedback controller implemented by the brain, and the integration of the velocity predictions *v*_*BMI*_ during brain control, respectively, as detailed in Equations (A11–A13 in Supplementary Material). The 2-degrees of freedom simulations are performed by evolving two independent systems for the x and y directions, respectively.

During normal simulations of pole and brain control, the process noise, ξ_*p*_, stems only from the noise in the control signal ξ_*u*_ (Equation A3 in Supplementary Material), i.e., ξ_*p*_ = *B*_*u*_ ξ_*u*_. Under assumption SA2, ξ_*u*_ is a white Gaussian noise, whose variance (note that in the single dimension case ξ_*u*_ is a scalar) is denoted by α^2^_*u*_. Hence the covariance matrix of the process noise can be expressed as

(9) $$\Omega^{p}=\alpha_{u}^{2}B_{u}{B^{\prime }}_{u}$$

The position and velocity of the cursor are measured via visual (V) feedback, while the position and velocity of the hand are measured via proprioceptive (P) feedback. Both visual and proprioceptive measurements are assumed to be corrupted by zero mean white Gaussian measurement noise and the covariance matrix of the combined measurement is denoted by Ω^*m*^.

#### 2.3.3. Brain model

Following current computational motor control theories, the brain is assumed to implement optimal state estimation and feedback control (Kuo, 1995; Wolpert et al., 1995; Todorov and Jordan, 2002). However, for simplicity, the computations are performed in the state space, rather than their neural representations. Neural recording is simulated by modeling neurons that encode the relevant signals including the estimated state and control signal (as detailed in Section 2.3.4). The brain model includes three parts as depicted in Figure 1A and further detailed in Appendix B (Supplementary Materials)(first two parts) and Section 2.3.4 (third part):

**Table:** 

A:	Observer that implements optimal state estimation by integrating the sensory feedback and internal model predictions. Under assumption (SA2), optimal estimation is achieved with Kalman filter (Wolpert et al., 1995; Stengel, 2012), as specified in Equation (B1 in Supplementary Material). During simulations of normal reaching movements (pole control) the internal model is assumed to be accurate and the Kalman filter is based on the actual covariance matrices of the process and measurements noise. By assumption (SA3), the same covariance matrices are also used for computing the Kalman filter when simulating brain control.
B:	Optimal controller that, under assumption (SA2), reduces to Linear Quadratic Gaussian (LQG) controller (Stengel, 2012), as specified in Equations (B5–B8 in Supplementary Material). The controller gains were computed off-line to minimize a standard cost function that includes penalty for the control effort and for deviations from the target, as specified in Equations (B2–B4 in Supplementary Material) (Kuo, 1995; Wolpert et al., 1995; Todorov and Jordan, 2002).
C:	Neural activity generator that converts the relevant signals i.e., the internally estimated cursor state and the control signal, into neural activity of a population of *N*_*n*_ simulated neurons using linear multi-variable tuning functions (as detailed in Section 2.3.4). This population of neurons simulates the population of neurons recorded during the BMI experiments, so their activity is used to train and drive the BMI filter during simulated pole and brain control, respectively, as detailed in 2.3.5. By assumption (SA4), spike-counts are generated as realizations of *doubly stochastic* Poisson processes.

#### 2.3.4. Neural activity generator

Many studies (Georgopoulos et al., 1986; Moran and Schwartz, 1999) demonstrated that during reaching movements the neural activity is linearly correlated with several motor parameters, including hand position, velocity, speed and gripping force. One of the main hypotheses in this work is that the neurons encode the estimated state and control signal, and that the observed correlations with the above motor parameters result from this encoding. Since we are interested in the statistics of the binned spike-count (in bins of 100 ms), we generate the binned spike-counts directly (instead of simulating the spike-train, and then binning the data). As outlined in Figure 1B, the binned spike-counts of each neuron are generated as a realization of a DSPP (Johnson, 1996; Dayan and Abbott, 2001) whose cumulative rate Γ is a linear function of the encoded signals. Specifically, the cumulative spike-rate of neuron *i* during the k-th bin, Γ^*i*^(*k*), is given by

(10) $$\Gamma^{i}(k)=max(\beta_{i,0}+\beta_{i}^{T}S(k),0)$$

where *S*(*k*) is the vector of encoded signals at time step *k*, β_*i*, 0_ is the bias of neuron *i* = 1 … *N*_*n*_ and β_*i*_ is the vector of its tuning weights.

Since the monkey is rewarded when the cursor is at the target, independent of the position of the hand, we assume that only the estimated cursor-state is encoded. Thus, the vector of encoded signals

(11) $$S={\left[\begin{array}{cccccccc} {\hat{p}}_{cx} & {\hat{p}}_{cy} & {\hat{v}}_{cx} & {\hat{v}}_{cy} & {\hat{S}}_{p} & u_{x} & u_{y} & u_{e} \end{array}\right]}^{\prime }$$

includes three 2-dimensional vectors: the estimated cursor position $\hat{p}={\left[\begin{array}{cc} {\hat{p}}_{cx} & {\hat{p}}_{cy} \end{array}\right]}^{\prime }$ velocity $\hat{v}={\left[\begin{array}{cc} {\hat{v}}_{cx} & {\hat{v}}_{cy} \end{array}\right]}^{\prime }$, and control signal $u={\left[\begin{array}{cc} u_{x} & u_{y} \end{array}\right]}^{\prime }$, and the magnitude of the last two (estimated speed $\hat{S}$_*p*_ = ||$\hat{v}$|| and control effort *u*_*e*_ = ||*u*||). The representation of the speed of movement in the neural activity, beyond the representation of the velocity vector, is well documented (Moran and Schwartz, 1999), and here we assume it reflects the encoding of the estimated speed $\hat{S}$_*p*_. Similarity, we assume that the magnitude of the control signal *u*_*e*_ is also encoded.

The tuning weights β_*i*_ to the kinematic parameters (position, velocity and speed) were selected to obtain PKM levels similar to the PKM observed in the BMI experiments during pole control. Specifically, the tuning weights for each kinematic parameter were selected from a uniform distribution whose variance was proportional to the destined PKM associated with that kinematic parameter and inversely proportionally to its variance. The tuning weights for the control signal were also selected from a uniform distribution, but its variance was adjusted to get the proper time-shift in the cross-correlation between the neural activity and the velocity, which are described in Section 3.1. The tuning vectors for the position, velocity and control parameters define the respective, and in general different, preferred directions (PD) and modulation depths.

The literature on cortical motor units suggests that neurons in different areas in the brain encode different information. The activity of M1 neurons has been shown to correlate with both the kinematics, including velocity, speed and direction of movement (Georgopoulos et al., 1982; Ashe and Georgopoulos, 1994) and with the applied forces (Evarts, 1968; Ashe, 1997; Todorov, 2000). PMd neurons are modulated mainly by the direction and amplitude of the movement (Messier and Kalaska, 2000; Hendrix et al., 2009). In agreement with those studies, the activity of M1 units recorded during the BMI experiments considered here were shown to predict well hand position, velocity and gripping force (73, 66, and 83% of variance, respectively), while the activity of the recorded PMd units predicted well hand position and velocity (48 and 46% of variance, respectively), but not the gripping force (29% of variance) (Carmena et al., 2003). Considering the above evidence, we simulate the activity of two sub-populations of neurons encoding either: (i) only the estimated cursor state (position, velocity and speed), or (ii) both the estimated cursor state and the control signal (vector and magnitude). Based on the evidence in the literature, we expect that the behavior of simulated neurons in those two sub-populations would be similar to the behavior of recorded PMd and M1 units, respectively, and hence refer to them as PMd-like and M1-like neurons. Such similarities are demonstrated in Section 3.1, where the cross-correlations between the movement velocity and either the recorded or simulated neural activity are compared.

#### 2.3.5. BMI filter

As in the BMI experiments (Carmena et al., 2003), the velocity in simulated brain control was predicted from the simulated spike-counts by a linear multi-lag multi-variable BMI filter

(12) $$v_{BMI}(k)=\theta_{0}+\sum_{j\;=\;1}^{N_{n}}\sum_{l\;=\;-L}^{0}\theta_{j}(l)N_{j}(k+l)$$

where *N*_*n*_ is the number of simulated neurons, *L* = 10 the number of lags used for prediction and *N*_*j*_(*k*) the spike count of neuron *j* in bin *k*. The filter parameters θ_0_ and θ_*j*_(*l*), (where *l* is the index of the lag) were computed using truncated Singular Value Decomposition (Zacksenhouse et al., 2007). Two BMI filters were trained to reconstruct the velocity in x and y, respectively, using the last 10 min of simulated pole control.

The performance of the experimental BMI filter (similarly trained on the last 10 min of pole control) was assessed by testing its predictions on a different section of pole control and was quantified by a coefficient of correlation of *R*($\hat{v}$, *v*) = 0.755 ± 0.02. We expect that this imperfect performance is critical for generating the observed changes in neural modulations. Hence, this performance served as a benchmark for tuning the number of neurons and the process noise in the simulation to obtain a similar performance (as detailed in Section 2.3.6).

#### 2.3.6. Model parameters

Model parameters are detailed in Table 1. The basic parameters of the hand model, visual measurement noise and cost function were taken from Todorov (2005) as noted in Table 1. The basic hand model was extended with a friction term to model the friction of the hand held pole. The basic cost function was augmented to describe the requirement for holding the cursor on the target as described in Appendix B (Equations B2–B4 in Supplementary Material) using the same weight parameters. The reaching time *t*_*f*_ (in Supplementary Equation B3) is uniformly distributed between 1.7-2.2 s in pole control and 2.4-6 s in brain control.

**Table 1:** **Model Parameters table**.

**Parameter**	**Description**	**Value**	**References**
Δ	Discretization time of the hand model	0.01[s]	Todorov, 2005
*m*	Hand mass	1[kg]	Todorov, 2005
γ	Coefficient of friction	7.7 [$\frac{\text{kg}}{\text{s}}$]	-
τ	Time constant of the muscle model	0.04[s]	Todorov, 2005
*w*_*v*_	Velocity cost function weight	0.2	Todorov, 2005
*w*_*f*_	Energy cost function weight	0.02	Todorov, 2005
*t*_*f*_	Reaching time in pole control (range)	1.7-2.2 [s]	Carmena et al., 2003
*t*_*f*_	Reaching time in brain control (range)	2.4-6 [s]	Carmena et al., 2003
$\sigma_{V_{p}}^{2}$	Variance of the visual (V) position measurement noise	(0.01m)^2^	Todorov, 2005
$\sigma_{V_{v}}^{2}$	Variance of the visual (V) velocity measurement noise	(0.1$\frac{\text{m}}{\text{s}}$)^2^	Todorov, 2005
$\sigma_{Pp}^{2}$	Variance of the proprioception (P) position measurement noise	*k*_*m*_(0.01m)^2^	Section 2.3.6/3.5
$\sigma_{Pv}^{2}$	Variance of the proprioception (P) velocity measurement noise	*k*_*m*_(0.1$\frac{\text{m}}{\text{s}}$)^2^	Section 2.3.6/3.5
*k*_*m*_	Proprioception measurement noise factor	10	Section 2.3.6/3.5
α_*u*_	Magnitude of the hand process noise	*k*_*u*_ · (4.6[*N*])	Section 2.3.6
*k*_*u*_	Hand process noise factor	0.1	Section 2.3.6
*N*_*n*_	Number of neurons	50	Section 2.3.6
*T*	Bin size	0.1[s]	Carmena et al., 2003

**Figure 2:**
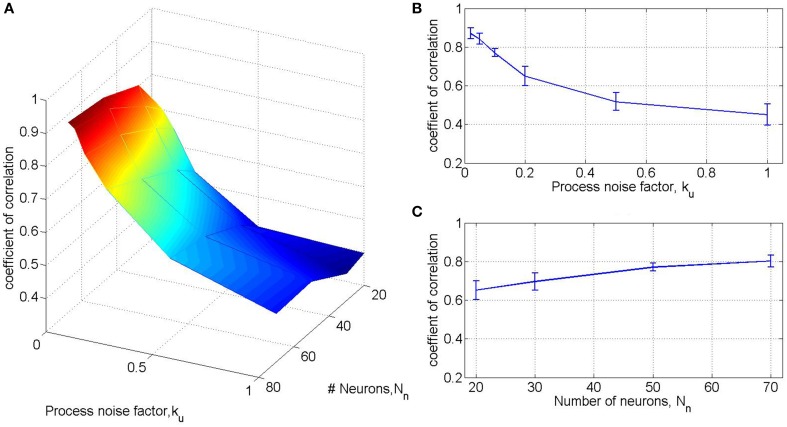
**BMI filter performance as a function of the number of neurons and the process noise factor**. Results are based on 15 simulated sessions of 20 min pole control, each with a different, randomly selected targets, but all with the same set of neural tuning weights. In each session the BMI filter was trained on the last 10 min and its performance was quantified by the coefficient of correlation between the actual and predicted velocity in the first 10 min. Each data point and error bar (in **B,C**) depict the average and standard deviation of the coefficient of correlations across the 15 sessions for a specific process noise factor *k*_*u*_ and total number of neurons *N*_*n*_. The effect of *k*_*u*_ is evaluated with *N*_*n*_ = 50 **(B)**, while the effect of *N*_*n*_ is evaluated with *k*_*u*_ = 0.1 **(C)**. A fixed 1:1 ratio is kept between the M1-like and PMd-like simulated neurons.

As detailed in our simplifying assumption SA2, the process noise during pole control is assumed to be signal independent, and its magnitude is quantified by of α_*u*_ in Equation (9). Initial simulations revealed that the performance of the experimental BMI filter, quantified by *R*($\hat{v}$, *v*) (Section 2.3.5), is highly sensitive to the process noise during simulated pole control, as shown in Figures 2A,B. The magnitude of the process noise was normalized by the equivalent signal-independent process noise suggested in Todorov (2005), signal-independent process noise suggested in the process noise factor defined as $k_{u}=\frac{\alpha_{u}}{4.6N}$. Figures 2A,C also depicts the effect of the total number of simulated neurons *N*_*n*_ on *R*($\hat{v}$, *v*), while the ratio between the two populations of simulated neurons, the M1-like and PMd-like neurons, was kept constant (1:1). The results in Figure 2 are based on 15 simulated sessions of 20 min pole control, each with a different, randomly selected targets, but all with the same set of neural tuning weights. In each session the coefficient of correlation between the actual and predicted velocity *R*($\hat{v}$, *v*) was computed from the first 10 min, while training was conducted on the last non-overlapping 10 min. Each data point and error bar (in (B-C)) depict the average and standard deviation of *R*($\hat{v}$, *v*) across the 15 sessions. Figure 2 suggests that with *N*_*n*_ = 50 and *k*_*u*_ = 0.1 the average performance of the BMI filter trained on simulated activity is *R*($\hat{v}$, *v*) = 0.77, close to the observed BMI filter performance. Hence, these are the default values used in all other standard simulations as indicated in Table 1.

While proprioceptive measurement noise has little effect on *R*($\hat{v}$, *v*) it has significant effect on the resulting changes in neural modulations. This effect is investigated in Section 3.5 and motivated the selection of a common 10-fold factor between the diagonal covariance matrices of the proprioceptive and visual measurements noise, as specified in Table 1. This proprioceptive noise factor, denoted by *k*_*m*_, was used during simulations of pole control and BCWHM, while during simulations of BCWOHM it was increased to infinity to reflect the lack of relevant proprioception measurements in this mode.

#### 2.3.7. Effect of process noise

Following our primary hypothesis that the observed changes in neural modulations can be explained in the framework OFC, we further hypothesize that these changes result from the higher process noise generated by the imperfect BMI filter that reconstructs the cursor velocity. This hypothesis is investigated both by simulations of noisy pole control, as outlined here, and by theoretical analysis in Section 2.4. Noisy pole control is simulated by using Equation (8) with the matrices associated with pole control (Equations A11–A13 in Supplementary Material), but with process noise that includes also the simulated effect of the BMI filter reconstruction error (as further explained in Appendix A2, Supplementary Material). Thus, instead of Equation (9) the covariance matrix of the process noise during noisy pole control simulations is

(13) $$\Omega_{noisy}^{p}=\alpha_{u}^{2}B_{u}B^{\prime }u+\alpha_{rec}^{2}B_{BMI}{B^{\prime }}_{BMI}$$

The first term represents the contribution of the noise in the control signal, and is the same as during regular pole control (Equation 9). The second term represents the additional noise introduced by the imperfect BMI filter, and its magnitude is quantified by α_*rec*_. The effect of varying the magnitude α_*rec*_ on neural modulations is investigated in Section 3.4.

### 2.4. Theoretical analysis of POM

As detailed in Section 2.3.7, we hypothesize that the observed changes in neural modulations following the transition to brain control result from the higher process noise introduced by the imperfect BMI filter. Here we investigate theoretically the effect of increasing process noise during simulations of normal reaching movements on POM defined theoretically in Equation (1), and show that under the assumptions of linear decoding and invariant internal model (see theoretical assumptions 1 and 2 below), the POM indeed increases with the process noise. The covariance matrix of the process noise, Ω^*p*^, is assumed to increase from a nominal low level Ω^*p*^_*L*_ to a high level Ω^*p*^_*H*_ such that the difference Ω^*p*^_*H*_ − Ω^*p*^_*L*_ is positive semi-definite (psd) (Horn and Johnson, 2012) and is denoted by Ω^*p*^_*H*_ − Ω^*p*^_*L*_ ≽ 0 or equivalently by Ω^*p*^_*H*_ ≽ Ω^*p*^_*L*_. Note that this analysis is general and does not rely on the specific structures of Ω^*p*^_*L*_ and Ω^*p*^_*H*_ suggested by Equations (9, 13), respectively.

We make the following simplifying theoretical assumptions (TA):

**Table:** 

TA1:	The cumulative spike-rate Γ encodes a linear combination of the estimated state. Since the optimal control signal is proportional to the estimated state, this includes also the control signal. However, non-linear functions, and in particular the estimated speed $\hat{S}$_*p*_ and the magnitude of the control effort *u*_*e*_, which are included in Equation (11), are not considered here.
TA2:	The internal model of the process noise does not change. This assumption follows directly from the simplifying assumption (SA3) that there is no adaptation to brain control.

Within the framework of state estimation and OFC, (TA1) implies that Γ can be expressed as the output of an extended linear system whose state $\tilde{x}={\left[\begin{array}{ccc} x_{k} & {\hat{x}}_{k|k} & 1 \end{array}\right]}^{T}$ includes both the actual and estimated states (of the hand and cursor). Following the details in Equations (C2–C5 in Supplementary Material), the extended system can be described as:

(14) $$\begin{array}{l} {\tilde{x}}_{k}={\tilde{A}}_{k}{\tilde{x}}_{k-1}+{\tilde{\xi }}_{k}\\ \Gamma_{k}={\tilde{H}}_{k}{\tilde{x}}_{k} \end{array}$$

where $\tilde{\xi }$ is the process noise of the extended system, which captures both the process noise of the actual system and the estimation error (Equation C4 in Supplementary Material). Since both are assumed to be white Gaussian noise, so is $\tilde{\xi }$, and its covariance matrix $E[{\tilde{\xi }}_{i}{\tilde{\xi }}_{j}]=\Omega_{i}^{\tilde{p}}\delta_{ij}$ is given by (see Equation C6 in Supplementary Material):

(15) $$\Omega_{k}^{\tilde{p}}=\left[\begin{array}{ccc} \Omega_{k}^{p} & 0 & 0\\ 0 & K_{k\;+\;1}H\Omega_{k}^{p}H^{T}K_{k\;+\;1}^{T}+K_{k\;+\;1}\Omega^{m}K_{k\;+\;1}^{T} & 0\\ 0 & 0 & 0 \end{array}\right]$$

Given (TA2), the Kalman gains *K*_*k*_ are optimized for the nominal system with the low process noise and are not updated to reflect the higher process noise.

**Proposition:** Let Γ^*L*^_*k*_ and Γ^*H*^_*k*_ be the outputs of two systems described by Equations (14, 15) with the same parameters except for the process noise that is either Ω^*p*^_*L*_ or Ω^*p*^_*H*_, respectively, where Ω^*p*^_*H*_ ≽ Ω^*p*^_*L*_. Let *POM*^*L*^ and *POM*^*H*^ be the POM (Equation 1) of the resulting spike counts generated by DSPPs from Γ^*L*^_*k*_ and Γ^*H*^_*k*_, respectively, then *POM*^*H*^ ≥ *POM*^*L*^.

**Proof:** Proposition C1 in Appendix C (Supplementary Material) proves that under the conditions of the proposition, the cumulative spike-rates Γ^*L*^_*k*_ and Γ^*H*^_*k*_ satisfy two conditions: (i) *E*_*m*_[Γ^*L*^_*k*_] = *E*_*m*_[Γ^*H*^_*k*_] and (ii) *E*_*m*_[(Γ^*H*^_*k*_)^2^] ≥ *E*_*m*_[(Γ^*L*^_*k*_)^2^], where *E*_*m*_[ · ] denotes ensemble average over different movements starting from the same distribution of initial conditions. Proposition C2 indicates that those two conditions assure that the POMs of the spike counts generated by DSPPs from Γ^*L*^_*k*_ and Γ^*H*^_*k*_, i.e., *POM*^*L*^ and *POM*^*H*^, respectively, satisfy *POM*^*H*^ ≥ *POM*^*L*^.

## 3. Results

The OFC model detailed in Section 2.3 was used to simulate 60 min sessions of BMI experiments equally divided into pole control, BCWHM and BCWOHM. Unless otherwise specified, new neural tuning weights and targets were randomly generated (as detailed in Sections 2.3.4 and 2.3.6) for each session. Since the performance of the BMI filter, quantified by *R*($\hat{v}$, *v*) (see Sections 2.3.5, 2.3.6), depends on the specific set of the neural tuning weights, only simulated sessions for which *R*($\hat{v}$, *v*) was between 0.65 and 0.85 were considered for further analysis (as further detailed in Section 3.2).

**Figure 3:**
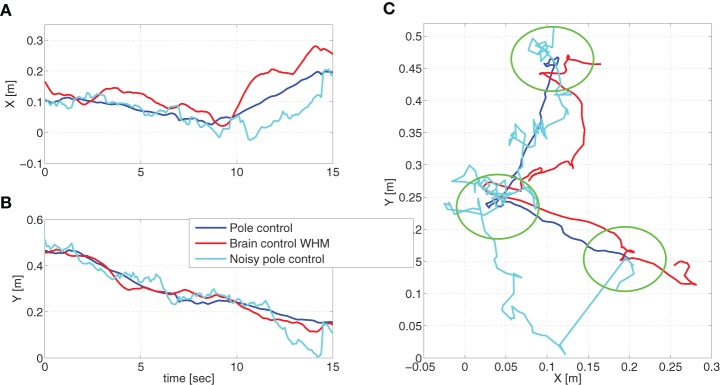
**Movement trajectories**. Representative traces of the cursor during simulated pole control, brain control and noisy pole control (with α_*rec*_ = 0.035). To facilitate comparison, the same set of targets (marked by green circles) and desired reaching times was used in these three simulations.

Simulated reaching movements under pole control resulted in an average speed profile that agrees well with the expected minimum jerk velocity profile (Flash and Hogan, 1985). Representative traces of the cursor during simulated pole control and BCWHM are shown in Figure 3. In order to facilitate comparison, the same sets of targets and desired reaching times were used. While in both phases the depicted traces reach the targets, they differ considerably, and are more variable in simulated BCWHM than in simulated pole control. In particular, the variance of the velocity (estimated over 2 min intervals) is significantly higher in simulated BCWHM than in simulated pole control (Wilcoxon rank sum test, *p* < 0.0001). This reproduces well the significant increase in the variance of the velocity from the experimental pole control to experimental BCWHM (Wilcoxon rank sum test, *p* < 0.0002).

The resulting cross correlations between the cursor velocity and neural activity generated during simulated pole control are investigated in Section 3.1 and compared with the cross-correlations derived from the BMI experiments. The main results regarding the changes in neural modulations are reported in Section 3.2. In Section 3.3 we investigate the effect of process noise on neural modulations during pole control, to support our hypothesis that increasing process noise introduced by the imperfect BMI filter contributes to the observed changes in neural modulations in brain control. Sections 3.4 and 3.5 evaluate the effect of the baseline measurement and process noise on estimated POM, respectively, and Section 3.6 investigates the effect of imperfect internal model. The $P\hat{O}M$ results described in this section and shown in the Figures were estimated from temporal statistics as detailed in Section 2.2.1 using Equation (3).

### 3.1. Cross correlation between cursor velocity and neural activity

**Figure 4:**
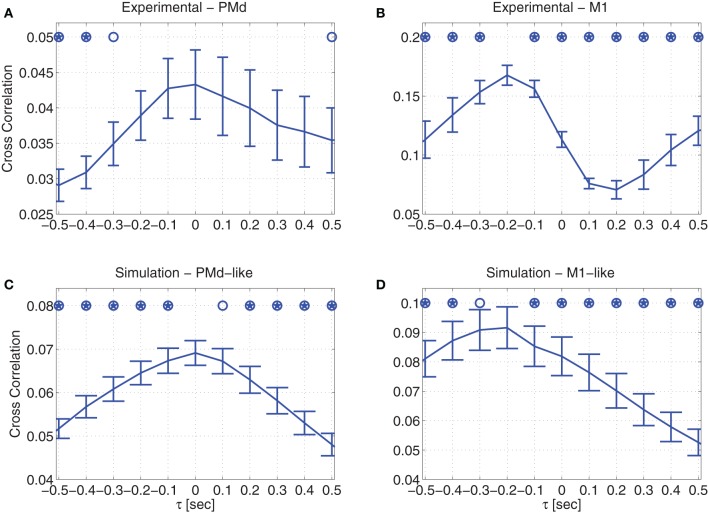
**Cross correlation between velocity and neural activity during pole control**. Averaged cross correlation of: **(A)** recorded data from PMd units **(B)** recorded data from M1 units **(C)** simulated PMd-like neurons **(D)** simulated M1-like neurons. Error bars depict standard deviations across non-overlapping 2-min intervals. Open circles and stars indicate cross-correlations that are significantly lower than the peak at 0 s in **(A,C)**, and at −0.2 s at **(B,D)** with significance level of either *p* = 0.05 (standard) or *p* = 0.005 (Bonferroni corrected for 10 multiple comparisons), respectively.

The average cross correlations between the recorded cursor velocity and the recorded neural activity of either PMd (Figure 4A) or M1 units (Figure 4B), demonstrate different temporal relationships. While there is no dominant time shift between the recorded neural activity of PMd neurons and the cursor velocity, the neural activity of M1 neurons clearly precede the velocity (Lebedev et al., 2005). The average cross correlations were computed by first calculating the cross-correlation for each unit from each non-overlapping 2-min interval, then averaging across units to get the ensemble-mean cross correlation, and finally averaging across intervals. Error bars depict the standard deviations of the ensemble-means across the non-overlapping 2-min intervals. Lags at which the cross correlation is significantly lower than the peak at either 0 s in (A) or −0.2 s in (B) are marked with open circles and stars, for significance level of p = 0.05 (standard) or p = 0.005 (Bonferroni corrected for 10 multiple comparisons), respectively (Wilcoxon rank sum test). It is evident that for M1 units, the peak at −0.2 s in Figure 4B is significantly higher than the cross-correlation at zero lag. In contrast, for PMd units, the cross-correlation at negative or positive lags are either significantly lower than the peak at 0 s or at least not significantly different (Figure 4A).

Similar temporal relationships are also observed in the simulations, depending on whether the simulated neurons encode only the estimated state or also the control signal. Figure 4C demonstrates that the activity of neurons that encode only the estimated state do not exhibit any dominant time shift with respect to the velocity, in agreement with the temporal relationship depicted by the recorded PMd units. As detailed in Section 2.3.4, this agreement is in line with the literature on PMd neural encoding (Messier and Kalaska, 2000; Hendrix et al., 2009), so we refer to these simulated neurons as PMd-like neurons.

In contrast, the activity of simulated neurons that encode both the estimated state and the control signal, precedes the simulated cursor velocity, as demonstrated in Figure 4D. In particular, the peak cross correlation at −0.2 s is significantly higher than the cross correlation at zero-lag. This is in agreement with the cross correlation depicted by the recorded M1 units. Given this agreement, and the literature on M1 neural encoding (Evarts, 1968; Kalaska et al., 1989; Todorov, 2000), we refer to these simulated neurons as M1-like neurons. The delay in the cursor velocity can be attributed to the effect of the muscles, which are modeled as a second order over-damped filter, and thus introduce a delay in the forces and eventually the movements that are generated in response to the control signal.

### 3.2. Neural modulations in simulated pole and brain control

**Figure 5:**
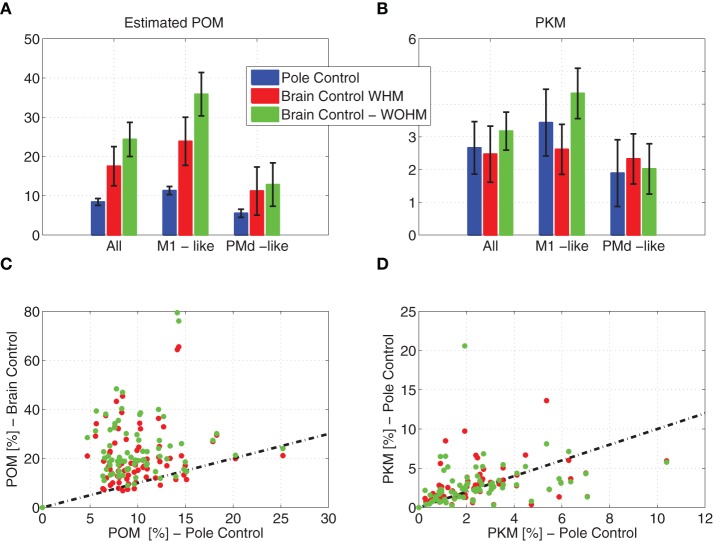
**Estimated POM and PKM of simulated neural activity**. Estimated POM and PKM were computed by first estimating the POM and PKM of the binned spike-counts of individual neurons in 2-min intervals, and then averaging across the relevant neurons and across the 10 intervals of each control mode. Mean estimated POM **(A)** and PKM **(B)** across all neurons and across neurons that encode only the estimated state (PMd-like) or also the control signals (M1-like). Error bars depict the standard deviations across the 10 intervals. Scatter plot of estimated POM **(C)** and PKM **(D)** in brain vs. pole control for 78 out of 100 sessions (each with a different set of tuning weights) in which the performance of the BMI filter satisfied 0.65 < *R*($\hat{v}$, *v*) < 0.85. Dashed lines depict the identity relationship.

Simulated neural activity generated by the model described in Section 2.3, based on the framework of OFC, successfully depicts the observed changes in neural modulations, as shown in Figure 5. Figures 5A,B depicts the results from a single session to facilitate direct comparison with the experimental results, while Figures 5C,D demonstrates the robustness of the results across a number of sessions with different sets of random tuning weights. As in Zacksenhouse et al. (2007), mean $P\hat{O}M$ and PKM were computed by first estimating the $P\hat{O}M$ and PKM from non-overlapping 2-min intervals of the binned spike-counts of individual neurons, then averaging across the relevant neurons to get the ensemble mean, and finally averaging across the 10 intervals of each control mode. Error bars in Figures 5A,B depict the standard deviations across the 10 intervals.

Figure 5 demonstrates that the $P\hat{O}M$ estimated from a single simulated session increases significantly when switching to brain control, with no matching increase in PKM. Focusing first on the detailed results from a single session in Figures 5A,B, the ensemble-mean $P\hat{O}M$ during simulated brain control is significantly higher than of ensemble-mean $P\hat{O}M$ during simulated pole control (Wilcoxon rank sum test, p = 0.001 for both BCWHM and BCWOHM), while the ensemble mean PKM did not differ significantly (p > 0.55). This behavior is evident in the simulated neural activity of both PMd-like and M1-like simulated neurons. This reproduces well the significant increase in the ensemble mean $P\hat{O}M$ from experimental pole control to BCWHM or BCWOHM (Wilcoxon rank sum test, *p* < 0.008), with no significant increase in the ensemble mean PKM (p>0.39).

The robustness of the changes in neural modulations following the transition to brain control was investigated by simulating 100 sessions, each with a different set of tuning weights. Pole control was simulated first, while brain control was conducted only in the 78 sessions in which the performance of the BMI filter was within the constraint described above (0.65 < *R*($\hat{v}$, *v*) < 0.85). The scatter plot in Figure 5C demonstrates that in these cases the $P\hat{O}M$ in BCWHM and BCWOHM are significantly higher than the $P\hat{O}M$ in pole control (Wilcoxon rank sum test, *p* < 10^−10^). In contrast, the scatter plot in Figure 5D demonstrates that the PKM in BCWHM and BCWOHM are not significantly higher than the PKM in pole control (*p* = 0.13 and *p* = 0.08, respectively).

### 3.3. Effect of process noise on neural modulations

Our main hypothesis is that the higher $P\hat{O}M$ in brain control, without matching increase in PKM, results from higher process noise introduced by the imperfect performance of the BMI filter. As detailed in Section 2.3.7, this hypothesis is investigated here by simulating noisy pole control in which the covariance of the process noise is characterized by Equation (13) instead of Equation (9). The additional term in Equation (13) describes the equivalent noise introduced by the imperfect BMI filter with magnitude that is quantified by α_*rec*_. Figure 3 demonstrates that indeed the cursor movements during noisy pole control (with α_*rec*_ = 0.035) are more similar to those during brain control than those during pole control. In particular, the variance of the velocity in the simulated trajectories generated with α_*rec*_ = 0.0035 was 88 ± 7 [($\frac{\text{cm}}{\text{sec}}$)^2^], comparable with the variance of the velocity in the simulated trajectories during BCWHM (75 ± 29 [($\frac{\text{cm}}{\text{sec}}$)^2^]), and significantly higher than the variance of the velocity in the simulated trajectories during pole control (Wilcoxon rank sum test, *p* < 0.0001).

**Figure 6:**
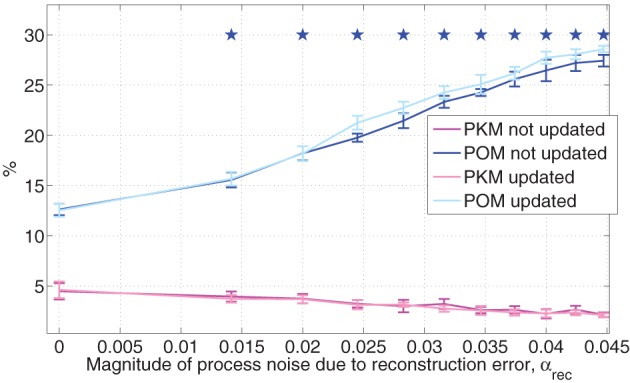
**Estimated POM and PKM during simulated noisy pole control**. Noisy pole control includes both the baseline process noise due to the control signal, and an additional process noise, simulating the contribution of the BMI filter reconstruction error, as quantified by the magnitude of α_*rec*_ in Equation (13). At each α_*rec*_ and for each adaptation option (updated or not), the average estimated POM and PKM were computed from a single 20 min session of simulated pole control as detailed in the caption of Figure 5. Standard deviations were computed across the 10 2-min intervals and stars indicate the estimated POM (for not-updated case) that are significantly larger than the estimated POM at the standard process noise (α_*rec*_ = 0) as determined by Wilcoxon rank sum test, with Bonferroni corrected significance level *p* = 0.005, corrected for 10 multiple comparisons. All sessions were performed with the same randomly selected targets and neural tuning weights.

The effects of α_*rec*_ on $P\hat{O}M$ and PKM are depicted in Figure 6 both when the internal model of the noise is not updated (in line with simplifying assumption SA3 that there is no adaptation) and when it is updated to match the actual process noise. All simulated sessions analyzed in Figure 6 were performed with the same set of randomly selected targets and neural tuning weights. At each α_*rec*_, the average $P\hat{O}M$ and PKM and their standard deviations were computed from a singe simulated session as detailed in Section 3.2.

Figure 6 demonstrates that as the process noise increases, due to increasing α_*rec*_, $P\hat{O}M$ increases while PKM does not change or even decreases. This is evident by the high positive (>0.99) and negative (< − 0.96) cross correlations between α_*rec*_ and either $P\hat{O}M$ or PKM, respectively. Furthermore, at all the noisy pole control cases analyzed in Figure 6, the $P\hat{O}M$ was significantly higher than the $P\hat{O}M$ with the standard process noise (i.e., with α_*rec*_ = 0, Wilcoxon rank sum test, with Bonferroni corrected significance level of *p* = 0.005, corrected for the 10 multiple comparisons), as marked by stars in Figure 6. The effect is similar whether the internal model of the process noise is updated to match the actual process noise or not. The results shown in Figure 6 are averaged $P\hat{O}M$ and PKM across all the simulated neurons, but similar trends were also observed when averaging across PMd-like and M1-like simulated neurons separately.

### 3.4. Sensitivity to baseline measurement noise

In this and the next section we investigate the sensitivity of $P\hat{O}M$ to the baseline covariance matrices of the different sources of noise, i.e., the covariance matrices of the measurement and process noise during pole control. Since the internal model is assumed to be well-adapted for pole control, these are also the covariance matrices of the internal model of the noise. Each sensitivity analysis is conducted with 15 sessions simulated with the same set of neurons but different sets of targets. This is equivalent to simulating longer sessions, which improves the accuracy of the estimated POM. In particular, the mean $P\hat{O}M$ across all the simulated neurons was estimated from each session and the average and standard deviations were computed across the 15 sessions.

The Kalman filter depends on the relative size of these covariance matrices, so the value of one of the elements can be kept constant. Furthermore, since we found that the $P\hat{O}M$ is insensitive to the ratio between visual position and velocity measurements noise, we kept the diagonal covariance matrix of the visual measurement noise fixed and equals to the one suggested in Todorov (2005).

**Figure 7:**
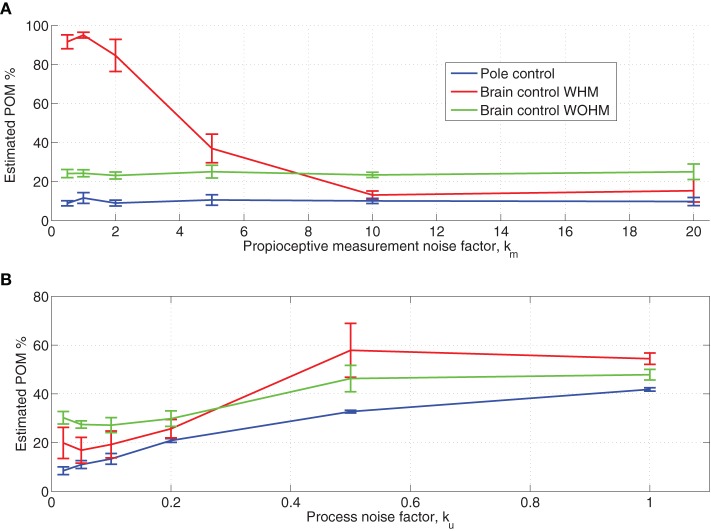
**Sensitivity of estimated POM to proprioceptive measurement noise (A) and magnitude of baseline process noise (B)**. The proprioceptive measurement noise factor *k*_*m*_ quantifies the ratio between the variance of the proprioceptive and the visual measurement noise (same factor for both position and velocity measurements). The process noise factor *k*_*u*_ quantifies the magnitude of hand process noise, as specified in Table 1. Each sensitivity analysis was conducted by simulating 15 sessions with the same set of neurons but different sets of targets. The mean estimated POM over the simulated neurons was computed for each session and the average and standard deviations across the 15 sessions are plotted.

Focusing first on the sensitivity of $P\hat{O}M$ to the baseline proprioceptive measurement noise, we found that the $P\hat{O}M$ is insensitive to the proprioceptive position measurement noise. Hence, Figure 7A depicts only the effect of a common scaling factor between the covariance matrices of the visual and proprioceptive measurements noise, i.e., the effect of the proprioceptive measurement noise factor *k*_*m*_ in Table 1. Figure 7A indicates that the $P\hat{O}M$ is sensitive to *k*_*m*_ only in BCWHM. When the covariance matrices of the proprioceptive and visual measurement noise are comparable (0.5 < *k*_*m*_ < 2), the $P\hat{O}M$ in simulated BCWHM is significantly larger than the $P\hat{O}M$ during the experimental BCWHM (Wilcoxon rank sum test, *p* < 0.004) and even larger than the $P\hat{O}M$ in BCWOHM, in contrast with the observed relationship in the BMI experiments (Zacksenhouse et al., 2007). When the proprioceptive measurement noise is 10-fold larger than the visual measurement noise (*k*_*m*_ = 10), the $P\hat{O}M$ in simulated BCWHM does not differ significantly from the $P\hat{O}M$ during the experimental BCWHM and is lower than the $P\hat{O}M$ in BCWOHM, as in the BMI experiments. Further increase in the the proprioceptive measurement noise has little effect on the $P\hat{O}M$ even in BCWHM. These observations motivated the selection of *k*_*m*_ = 10 as the default factor in all other simulations (Table 1).

The lack of sensitivity to proprioceptive measurement noise during pole control can be attributed to the perfect internal model and low baseline process noise, which cause state-estimation to be based mainly on the internal model. The high $P\hat{O}M$ during BCWHM when the baseline proprioceptive and visual measurements noise are comparable can be attributed to the resulting similar Kalman gains for those two modalities. Due to the imperfect BMI filter, the estimation measurement error in brain control is high, and during BCWHM the proprioceptive measurement may differ from the visual measurement. When both modalities are given similar Kalman gains, the deviation between the proprioceptive and visual measurements contributes to higher variance in the resulting estimated state. Finally, during BCWOHM the proprioceptive measurement noise is increased to infinity to model the lack of relevant proprioceptive feedback in this mode, and hence the $P\hat{O}M$ in this mode does not depend on the baseline proprioceptive measurement noise.

### 3.5. Sensitivity to baseline process noise

During regular pole control, the covariance of the process noise is described by Equation (9) and its magnitude is quantified by α_*u*_, or alternatively by the process noise factor *k*_*u*_, as described in Section 2.3.6 and Table 1. As noted in Section 2.3.6 and Figure 2, adequate performance of the BMI filter in the simulated BMI sessions can be achieved with *k*_*u*_ = 0.1. Here we investigate the effect of *k*_*u*_ on $P\hat{O}M$.

Figure 7B depicts the changes in the $P\hat{O}M$ as a function of the process noise factor *k*_*u*_. It is apparent that $P\hat{O}M$ in brain control is always higher than $P\hat{O}M$ in pole control, in agreement with the observed changes in the $P\hat{O}M$ during the BMI experiments. However, for *k*_*u*_ = 1, the $P\hat{O}M$ in each of control mode is too large compared with the experimentally observed $P\hat{O}M$ (Wilcoxon rank sum test, *p* < 0.002 for each control mode). This further supports the selection of a smaller process noise factor, and in particular *k*_*u*_ = 0.1, made in section 2.3.6 based on the performance of the BMI filter. For this process noise, the relation between the $P\hat{O}M$ in BCWHM versus BCWOHM agrees well with the experimental observations.

Figure 7B also shows that $P\hat{O}M$ increases with the magnitude of the process noise. This is in agreement with the results described in Section 3.4, though in that section the magnitude of the process noise described by Equation (13) was manipulated by changing of α_*rec*_ instead of α_*u*_ (via *k*_*u*_).

### 3.6. Internal model variations

**Figure 8:**
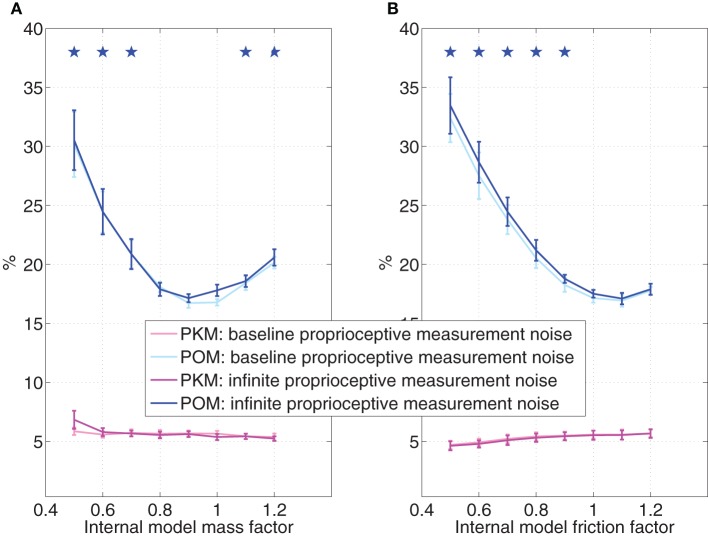
**Effect of internal model variations during pole control**. The value of the mass **(A)** or the coefficient of friction **(B)** in the internal model is normalized to the value of the actual parameter. Simulations are conducted with either the baseline proprioceptive measurement noise characteristic of pole control or infinite proprioceptive measurement noise characteristic of BCWOHM. At each parameter value, and for each level of the proprioceptive measurement noise, the average estimated POM and PKM were computed from a single 20 min session of simulated pole control as detailed in the caption of Figure 5. Standard deviations were computed across the 10 2-min intervals and stars indicate estimated POMs that are significantly larger than the estimated POM at the nominal parameter (internal model factor = 1), as determined by Wilcoxon rank sum test with Bonferroni corrected significance level *p* = 0.007, corrected for 7 multiple comparisons. All sessions were performed with the same randomly selected targets and neural tuning weights.

The simulations presented so far were based on simplifying assumption SA3, so the internal model did not change due to transition to brain control. Since the monkey stopped moving its hand in BCWOHM, the internal model may change to account for the lack of hand movements. Figure 8 evaluates the effect of changing the parameters of the internal model, and in particular the mass (Figure 8A), or the coefficient of friction (Figure 8B), during pole control while keeping the external model the same. In both cases, the x-axis is the normalized parameter of the internal model (mass or coefficient of friction) relative to the nominal value of the parameter of the external model. The effect is evaluated both with the baseline proprioceptive measurement noise characteristic of pole control and BCWHM, and with infinite proprioceptive measurement noise characteristic of BCWOHM. All simulated sessions analyzed in Figure 8 were performed with the same set of randomly selected targets and neural tuning weights, but not the same set used in other sections. At each parameter value, and for each level of the proprioceptive measurement noise, the average $P\hat{O}M$ and PKM in Figure 8 and their standard deviations were computed from a single simulated session as detailed in Section 3.2.

Figure 8 indicates that when the internal model deviates from the external model, the $P\hat{O}M$ increases significantly, while the PKM does not change significantly or even decease over most of the range. Parameter values at which the $P\hat{O}M$ (generated in simulations with infinite process noise) is significantly higher than the $P\hat{O}M$ at the nominal parameter are marked by stars (Wilcoxon rank sum test with Bonferroni corrected significance level *p* = 0.007, corrected for 7 multiple comparisons). The effect of increasing the covariance matrix of the proprioceptive measurement noise to infinity, as in BCWOHM, is negligible. Hence, changes in the parameters of the internal model may further contribute the observed change in $P\hat{O}M$ upon switching to brain control, and especially to BCWOHM.

## 4. Discussion

### 4.1. Modeling

The implemented OFC model successfully moves the hand and cursor in simulated pole control to reach randomly selected targets. Neural activity is generated to simulate neural recording and perform brain control. The simulated neurons are assumed to encode the signals that are relevant for OFC, i.e., the estimated state and the control signal, and spike-counts are generated as doubly stochastic Poisson processes. The model parameters were taken from the literature or selected to match the experimental observations. In particular, the magnitude of the process noise and the number of neurons were selected so the performance of the BMI filter trained on the simulated neural activity matches the performance of the BMI filter trained on the recorded neural activity. The resulting model successfully generates the main phenomena that it was developed to explain: the POM of the neural activity in brain control is higher than the POM in pole control with no matching increase in PKM.

OFC involves different computational tasks that can be mapped to different brain regions. In particular, it has been suggested (Shadmehr and Krakauer, 2008) that expected costs and rewards are evaluated by the basal ganglia, the forward model is implemented by the cerebellum, and the predicted sensory feedback is combined with the actual sensory feedback at the parietal cortex to estimate the state. Given the estimated state, the pre-motor and primary motor cortices are hypothesized to generate the control signals associated with visual and proprioceptive feedback, respectively. As mentioned above (Introduction and Section 2.3.3) the OFC is simulated in the state space, so all these computational tasks are included regardless of where they are implemented in the brain. In contrast, neural activity is generated to simulate neural recording from M1 and PMd only, since those are the main brain regions recorded in the BMI experiments considered here.

### 4.2. Neural encoding

Neural encoding is an open and important research question. Common strategies to investigate this issue involve quantifying the correlation between the neural activity and different relevant signals, or assessing how well those signals can be predicted by the neural activity. However, those strategies are usually restricted to measurable signals. In particular, many studies investigated the correlation between the activity of cortical motor units and different kinematics and kinetics signals (Georgopoulos et al., 1984, 1986, 1992; Kalaska et al., 1989; Ashe and Georgopoulos, 1994; Ashe, 1997; Moran and Schwartz, 1999; Paninski et al., 2004; Kalaska, 2009). Since correlations do not imply causality, the observed correlations with any of the measurable signals do not imply that the neurons indeed encode these signals. Instead, the neurons may encode internal, hidden signals, which are also correlated with the measurable signals (Zacksenhouse et al., 2014) including, for example, muscle activation patterns (Todorov, 2000).

Here we suggest that cortical motor neurons encode signals that are relevant for performing state estimation and control, i.e., the estimated state and the control signal. Since these signals are internal signals generated by the brain, direct correlation cannot be evaluated. Nevertheless, during normal reaching movements, the actual movement should agree well with the estimated state, and thus the neural activity would appear to be correlated with the movement. However, in other conditions, and in particular during brain control, the estimated state may deviate from the measured state. This work suggests that the higher POM during brain control with no matching increase in PKM may arise from such deviations.

We noted that the neural activity recorded from M1 and PMd units exhibited different cross-correlations with the velocity. In particular, the average cross-correlations between the velocity and the recorded neural activity during pole control indicate that there is no time-shift between the neural activity of PMd units and the cursor velocity, while the neural activity of M1 neurons precedes the velocity. These temporal relationships are reproduced well by simulated neurons that encode only the estimated state or also the control signal, respectively. The time shift in the latter case can be ascribed to the delay in the response of the muscles. Hence, the different types of observed cross-correlations, with and without time shift, can be attributed to encoding different signals within the framework OFC, including and excluding the control signal, respectively. Encoding the control signal in M1 but not in PMd neurons agrees well with evidence that the activity of PMd neurons is modulated mainly by the direction and magnitude of movements while the activity of M1 neurons is correlated also with the forces (Evarts, 1968; Kalaska et al., 1989). Further support is provided by previous investigation of the BMI experiments considered here, which indicated that PMd neurons can predict well the position and velocity but not the force, while M1 neurons can predict well the force too (Carmena et al., 2003). While this specific distinction between M1 and PMd neurons may result from the specific sample of units recorded during the BMI experiments considered here, it is well reproduced by the simulated model based on OFC.

### 4.3. Process noise

Our main hypothesis is that the observed increase in POM following the transition to brain control reflects increasing variance of the encoded signals due to higher process noise. The latter is assumed to occur in brain control due to the limited accuracy of the BMI filter. This hypothesis was proved theoretically and by simulations. Theoretical proof was limited to the case when the estimated process noise in the internal model is not updated, and the neurons encode linear combinations of the estimated state. The simulations demonstrate that increasing process noise during pole control results in higher POM even when neurons encode non-linear functions of the estimated state (e.g., speed and magnitude of the control signal) and regardless of whether the internal model is updated or not.

### 4.4. Implications for BMI

The proposed model attributes the increase in POM following the transition to brain control to the higher process noise introduced by the imperfect performance of the BMI filter. This suggests that the POM is a proxy for the performance of the BMI filter: High POM indicates that the BMI filter performs poorly while low POM indicates that the BMI filter performs well. As a proxy signal for the performance of the BMI filer, the POM could be used to adapt the BMI filter using reinforcement learning (DiGiovanna et al., 2009; Mahmoudi et al., 2013), especially in cases when an error signal cannot be determined (Bensmaia and Miller, 2014). Furthermore, the proposed model developed here, provides a framework for investigating this and other suggestion for improving BMIs. Finally, the proposed model can be used to investigate other changes that occur following the transition to brain control and in particular the changes in neural tuning (Lebedev et al., 2005).

### 4.5. Alternative hypotheses

Our main hypothesis is that the observed changes in neural modulations following the transition to brain control are related to changes in the process and measurement noise induced by the transition, and in particular, the increase in process noise due to the imperfect BMI filter. OFC provides a coherent and concise framework for relating the changes in process and measurement noise to changes in the estimated state and control signals, and finally to changes in neural modulations. However, the key elements are state estimation and the encoding of the estimated state, so other models that include those components may produce similar results. In particular, state estimation and control may not be optimal, but only satisficing (Simon, 1956; Brown et al., 2004). The OFC framework was adopted here since optimization constrains the parameters of the estimation filter and the gains of the control law, resulting in fewer free parameters to be tuned, thus granting the resulting model higher credibility.

An alternative hypothesis that may contribute to the changes in neural modulations involve changes in the internal model. As was shown in Section 3.6, deviations between the internal model and the external system, and in particular deviations in the mass or the coefficient of friction, result in higher $P\hat{O}M$ with no matching increase in PKM. Thus, changes in the internal model of the mass or coefficient of friction, may contribute to the increase in $P\hat{O}M$ beyond what is attributed to the increase in process noise. Since the transition to BCWOHM involves stopping hand movements, it is conceivable that it is associated with a switch to a different internal model, with lower mass and coefficient of friction. However, the change in the internal model is not justified in the transition to BCWHM, when the monkey continues to make hand movements.

Alternative frameworks for explaining the changes in neural modulations may be provided by intermittent predictive control (Doeringer and Hogan, 1998; Gawthrop et al., 2011) and active inference (Friston et al., 2010, 2011). Intermittent predictive control alleviates the computational load of prediction that is necessary to address time delays (Doeringer and Hogan, 1998; Gawthrop et al., 2011). Due to the imperfect BMI filter, the transition to brain control is expected to result in more frequent updates of the predictor, and hence in higher variability in the predicted state. Since intermittent predictive control is based on OFC, this is an extension of the current model, and will be considered in future work. Indeed it could be argued that due to the inaccurate movements of the cursor during brain control, the monkey is making explorative movements to adapt to the new environment. While the explorative movements may contribute to higher POM, they are expected to also increase the PKM, in contrast with the observation.

In summary, we demonstrated that the observed changes in neural modulations following the transition to brain control can be successfully explained under the assumption that the neurons encode the estimated state and control signal. To be concrete we used the framework of OFC, but similar results are expected even if the controller is not optimal, as long as it relies on state estimation that deteriorates upon switching to brain control.

### Conflict of interest statement

The authors declare that the research was conducted in the absence of any commercial or financial relationships that could be construed as a potential conflict of interest.

## References

[B1] Ashe J. (1997). Force and the motor cortex. Behav. Brain Res. 87, 255–269. 10.1016/S0166-4328(97)00752-3 9331494

[B2] Ashe J. Georgopoulos A. P. (1994). Movement parameters and neural activity in motor cortex and area 5. Cereb. Cortex 4, 590–600. 10.1093/cercor/4.6.590 7703686

[B3] Bensmaia S. J. Miller L. E. (2014). Restoring sensorimotor function through intracortical interfaces: progress and looming challenges. Nat. Rev. Neurosci. 15, 313–325. 10.1038/nrn3724 24739786 PMC12278825

[B4] Brown E. N. Kass R. E. Mitra P. P. (2004). Multiple neural spike train data analysis: state-of-the-art and future challenges. Nat. Neurosci. 7, 456–461. 10.1038/nn1228 15114358

[B5] Carmena J. M. Lebedev M. A. Crist R. E. O'Doherty J. E. Santucci D. M. Dimitrov D. F.. (2003). Learning to control a brain-machine interface for reaching and grasping by primates. PLoS Biol. 1:E42. 10.1371/journal.pbio.0000042 14624244 PMC261882

[B6] Chang Y. H. Chen M. Shanechi M. Carmena J. M. Tomlin C. (2014). A design of neural decoder by reducing discrepancy between Manual Control (MC) and Brain Control (BC), in 2014 European Control Conference (ECC) (Strasbourg), 516–521.

[B7] Churchland A. K. Kiani R. Chaudhuri R. Wang X.-J. Pouget A. Shadlen M. N. (2011). Variance as a signature of neural computations during decision making. Neuron 69, 818–831. 10.1016/j.neuron.2010.12.037 21338889 PMC3066020

[B8] Crevecoeur F. Scott S. H. (2013). Priors engaged in long-latency responses to mechanical perturbations suggest a rapid update in state estimation. PLoS Comput. Biol. 9:e1003177 10.1371/journal.pcbi.1003177 23966846 PMC3744400

[B9] Cunningham J. P. Nuyujukian P. Gilja V. Chestek C. A. Ryu S. I. Shenoy K. V. (2011). A closed-loop human simulator for investigating the role of feedback control in brain-machine interfaces. J. Neurophysiol. 105, 1932–1949. 10.1152/jn.00503.2010 20943945 PMC3075301

[B10] Dayan P. Abbott L. F. (2001). Theoretical Neuroscience. Cambridge, MA: MIT Press.

[B11] DiGiovanna J. Mahmoudi B. Fortes J. Principe J. C. Sanchez J. C. (2009). Coadaptive brain–machine interface via reinforcement learning. Biomed. Eng. IEEE Trans. 56, 54–64. 10.1109/TBME.2008.926699 19224719

[B12] Doeringer J. A. Hogan N. (1998). Intermittency in preplanned elbow movements persists in the absence of visual feedback. J. Neurophysiol. 80, 1787–1799. 9772239 10.1152/jn.1998.80.4.1787

[B13] Evarts E. V. (1968). Relation of pyramidal tract activity to force exerted during voluntary movement. J. Neurophysiol. 31, 14–27. 4966614 10.1152/jn.1968.31.1.14

[B14] Flash T. Hogan N. (1985). The coordination of arm movements: an experimentally confirmed mathematical model. J. Neurosci. 5, 1688–1703. 4020415 10.1523/JNEUROSCI.05-07-01688.1985 PMC6565116

[B15] Friston K. Mattout J. Kilner J. (2011). Action understanding and active inference. Biol. Cybern. 104, 137–160. 10.1007/s00422-011-0424-z 21327826 PMC3491875

[B16] Friston K. J. Daunizeau J. Kilner J. Kiebel S. J. (2010). Action and behavior: a free-energy formulation. Biol. Cybern. 102, 227–260. 10.1007/s00422-010-0364-z 20148260

[B17] Gawthrop P. Loram I. Lakie M. Gollee H. (2011). Intermittent control: a computational theory of human control. Biol. Cybern. 104, 31–51. 10.1007/s00422-010-0416-4 21327829

[B18] Geisler W. Albrecht D. (1995). Bayesian analysis of identification performance in monkey visual cortex: nonlinear mechanisms and stimulus certainty. Vis. Res. 35, 2723–2730. 10.1016/0042-6989(95)00029-Y 7483312

[B19] Georgopoulos A. Caminiti R. Kalaska J. (1984). Static spatial effects in motor cortex and area 5: quantitative relations in a two-dimensional space. Exp. Brain Res. 54, 446–454. 10.1007/BF00235470 6723864

[B20] Georgopoulos A. P. Ashe J. Smyrnis N. Taira M. (1992). The motor cortex and the coding of force. Science 256, 1692–1695. 10.1126/science.256.5064.1692 1609282

[B21] Georgopoulos A. P. Kalaska J. F. Caminiti R. Massey J. T. (1982). On the relations between the direction of two-dimensional arm movements and cell discharge in primate motor cortex. J. Neurosci. 2, 1527–1537. 7143039 10.1523/JNEUROSCI.02-11-01527.1982 PMC6564361

[B22] Georgopoulos A. P. Schwartz A. B. Kettner R. E. (1986). Neuronal population coding of movement direction. Science 233, 1416–1419. 10.1126/science.3749885 3749885

[B23] Harris C. M. Wolpert D. M. (1998). Signal-dependent noise determines motor planning. Nature 394, 780–784. 10.1038/29528 9723616

[B24] Hendrix C. M. Mason C. R. Ebner T. J. (2009). Signaling of grasp dimension and grasp force in dorsal premotor cortex and primary motor cortex neurons during reach to grasp in the monkey. J. Neurophysiol. 102, 132–145. 10.1152/jn.00016.2009 19403752 PMC2712255

[B25] Horn R. A. Johnson C. R. (2012). Matrix Analysis. Cambridge, MA: Cambridge University Press 10.1017/CBO9781139020411

[B26] Johnson D. H. (1996). Point process models of single-neuron discharges. J. Comput. Neurosci. 3, 275–299. 10.1007/BF00161089 9001973

[B27] Jordan M. I. Rumelhart D. E. (1992). Forward models: supervised learning with a Distal teacher. Cogn. Sci. 16, 307–354. 10.1207/s15516709cog1603_1

[B28] Kalaska J. F. (2009). From intention to action: motor cortex and the control of reaching movements, in Progress in Motor Control, ed Sternad D. (Springer US), 139–178. 10.1007/978-0-387-77064-2_8 19227499

[B29] Kalaska J. F. Cohen D. Hyde M. L. Prud'Homme M. (1989). A comparison of movement direction-related versus load direction-related activity in primate motor cortex, using a two-dimensional reaching task. J. Neurosci. 9, 2080–2102. 2723767 10.1523/JNEUROSCI.09-06-02080.1989 PMC6569743

[B30] Kawato M. Furukawa K. Suzuki R. (1987). A hierarchical neural-network model for control and learning of voluntary movement. Biol. Cybern. 57, 169–185. 10.1007/BF00364149 3676355

[B31] Kuo A. D. (1995). An optimal control model for analyzing human postural balance. Biomed. Eng. IEEE Trans. 42, 87–101. 10.1109/10.362914 7851935

[B32] Lebedev M. A. Carmena J. M. O'Doherty J. E. Zacksenhouse M. Henriquez C. S. Principe J. C.. (2005). Cortical ensemble adaptation to represent velocity of an artificial actuator controlled by a brain-machine interface. J. Neurosci. 25, 4681–4693. 10.1523/JNEUROSCI.4088-04.2005 15888644 PMC6724781

[B33] Mahmoudi B. Pohlmeyer E. A. Prins N. W. Geng S. Sanchez J. C. (2013). Towards autonomous neuroprosthetic control using hebbian reinforcement learning. J. Neural Eng. 10, 66005. 10.1088/1741-2560/10/6/066005 24100047

[B34] Messier J. Kalaska J. F. (2000). Covariation of primate dorsal premotor cell activity with direction and amplitude during a memorized-delay reaching task. J. Neurophysiol. 84, 152–165. 10899193 10.1152/jn.2000.84.1.152

[B35] Miall R. Wolpert D. M. (1996). Forward models for physiological motor control. Neural Netw. 9, 1265–1279. 10.1016/S0893-6080(96)00035-4 12662535

[B36] Moran D. W. Schwartz A. B. (1999). Motor cortical representation of speed and direction during reaching. J. Neurophysiol. 82, 2676–2692. 10561437 10.1152/jn.1999.82.5.2676

[B37] Nicolelis M. A. (2001). Actions from thoughts. Nature 409, 403–407. 10.1038/35053191 11201755

[B38] Paninski L. Fellows M. R. Hatsopoulos N. G. Donoghue J. P. (2004). Spatiotemporal tuning of motor cortical neurons for hand position and velocity. J. Neurophysiol. 91, 515–532. 10.1152/jn.00587.2002 13679402

[B39] Schwartz A. B. (2004). Cortical neural prosthetics. Annu. Rev. Neurosci. 27, 487–507. 10.1146/annurev.neuro.27.070203.144233 15217341

[B40] Shadmehr R. Krakauer J. W. (2008). A computational neuroanatomy for motor control. Exp. Brain Res. 185, 359–381. 10.1007/s00221-008-1280-5 18251019 PMC2553854

[B41] Shanechi M. M. Williams Z. M. Wornell G. W. Hu R. C. Powers M. Brown E. N. (2013a). A real-time brain-machine interface combining motor target and trajectory intent using an optimal feedback control design. PLoS ONE 8:e59049 10.1371/journal.pone.0059049 23593130 PMC3622681

[B42] Shanechi M. M. Wornell G. W. Williams Z. M. Brown E. N. (2013b). Feedback-controlled parallel point process filter for estimation of goal-directed movements from neural signals. Neural Syst. Rehabil. Eng. IEEE Trans. 21, 129–140. 10.1109/TNSRE.2012.2221743 23047892

[B43] Shenoy K. V. Sahani M. Churchland M. M. (2013). Cortical control of arm movements: a dynamical systems perspective. Annu. Rev. Neurosci. 36, 337–359. 10.1146/annurev-neuro-062111-150509 23725001

[B44] Simon H. A. (1956). Rational choice and the structure of the environment. Psychol. Rev. 63, 129. 10.1037/h0042769 13310708

[B45] Snyder D. L. (1975). Random Point Processes. New York, NY: J. Willey & Sons.

[B46] Stengel R. F. (2012). Optimal Control and Estimation. London: Courier Dover Publications.

[B47] Taylor D. M. Tillery S. I. H. Schwartz A. B. (2002). Direct cortical control of 3d neuroprosthetic devices. Science 296, 1829–1832. 10.1126/science.1070291 12052948

[B48] Todorov E. (2000). Direct cortical control of muscle activation in voluntary arm movements: a model. Nat. Neurosci. 3, 391–398. 10.1038/73964 10725930

[B49] Todorov E. (2005). Stochastic optimal control and estimation methods adapted to the noise characteristics of the sensorimotor system. Neural Comput. 17, 1084–1108. 10.1162/0899766053491887 15829101 PMC1550971

[B50] Todorov E. Jordan M. I. (2002). Optimal feedback control as a theory of motor coordination. Nat. Neurosci. 5, 1226–1235. 10.1038/nn963 12404008

[B51] Tolhurst D. J. Movshon J. Dean A. (1983). The statistical reliability of signals in single neurons in cat and monkey visual cortex. Vis. Res. 23, 775–785. 10.1016/0042-6989(83)90200-6 6623937

[B52] Wolpert D. Ghahramani Z. Jordan M. (1995). Are arm trajectories planned in kinematic or dynamic coordinates? An adaptation study. Exp. Brain Res. 103, 460–470. 10.1007/BF00241505 7789452

[B53] Wolpert D. M. Ghahramani Z. (2000). Computational principles of movement neuroscience. Nat. Neurosci. 3, 1212–1217. 10.1038/81497 11127840

[B54] Wu W. Gao Y. Bienenstock E. Donoghue J. P. Black M. J. (2006). Bayesian population decoding of motor cortical activity using a Kalman filter. Neural Comput. 18, 80–118. 10.1162/089976606774841585 16354382

[B55] Zacksenhouse M. Lebedev M. A. Carmena J. M. O'Doherty J. E. Henriquez C. Nicolelis M. a. L. (2007). Cortical modulations increase in early sessions with brain-machine interface. PLoS ONE 2:e619. 10.1371/journal.pone.0000619 17637835 PMC1919433

[B56] Zacksenhouse M. Lebedev M. A. Nicolelis M. A. L. (2014). Signal-independent timescale analysis (SITA) and its application for neural coding during reaching and walking. Front. Comput. Neurosci. 8:91. 10.3389/fncom.2014.00091 25191263 PMC4137543

[B57] Zacksenhouse M. Nemets S. (2008). Strategies for neural ensemble data analysis for brain-machine interface (BMI) applications, in Methods Neural Ensemble Record, 2nd Edition, Chapter 4, ed Nicolelis M. (Boca Raton, FL: CRC Press), 57–82. 10.1201/9781420006414.ch4 21204438

