# Erratum: Optimal feedback control successfully explains changes in neural modulations during experiments with brain-machine interfaces

**DOI:** 10.3389/fnsys.2015.00137

**Published:** 2015-09-29

**Authors:** Frontiers Production Office

**Affiliations:** Frontiers Production Office, FrontiersLausanne, Switzerland

**Keywords:** brain-machine interfaces, neural modulations, optimal feedback control, computational motor control, process noise

Reason for Erratum:

Due to a typesetting error, the reviewer Frédéric Crevecoeur was inadvertently removed from the final published article. The publisher apologizes for this mistake.

This error does not change the scientific conclusions of the article in any way.

The original article has been updated.

