# Supplementary material for: Optimal feedback control successfully explains changes in neural modulations during experiments with brain-machine interfaces
Source: Front Syst Neurosci. 2015 May 19;9:71. doi: 10.3389/fnsys.2015.00071 (PMC4436824; doi:10.3389/fnsys.2015.00071)
Supplement: Supplementary file 1 [file Presentation1.PDF]

APPENDIX

A HAND AND CURSOR MODEL

A.1 Hand model For simplicity, we present the model for a single dimension (x or y), while actual simulations were conducted with two independent dimensions. Following [49] the hand is modeled as a point mass driven by an over damped second order muscle model with time constants $\tau_1 = \tau_2 = \tau$ that responds to the control signal from the brain [53]. An additional friction term is introduced to model the friction of the hand held pole. The combined dynamics of the hand and the muscle can be represented as a continuous- time state space model

$$\dot{x}_h(t) = A_{h,cnt}x_h(t) + B_{h,cnt}(u(t) + \xi_u(t)) \tag{A1}$$

where, the hand state in each dimension, $x_h = [p_h \quad v_h \quad f \quad g]$, includes the position, p_h and velocity, v_h, of the hand (in a single dimension), the force generated by the muscle f and the internal state of the muscle g; and $\xi_u(t)$ is the noise associated with the control signal $u(t)$. The system matrices are:

$$A_{h,cnt} = \begin{bmatrix} 0 & 1 & 0 & 0 \\ 0 & -\frac{\gamma}{m} & \frac{1}{m} & 0 \\ 0 & 0 & -\frac{1}{\tau_1} & -\frac{1}{\tau_2} \\ 0 & 0 & 0 & -\frac{1}{\tau_1} \end{bmatrix}; B_{h,cnt} = \begin{bmatrix} 0 \\ 0 \\ 0 \\ \frac{1}{\tau_1} \end{bmatrix} \tag{A2}$$

Where m is the mass and γ the coefficient of friction. Assuming zero order hold for the control signal $u(t)$ the differential equations can be discretized at intervals $\Delta = 0.01sec$ and expressed as a difference system (where the index k denotes the k-th sample at 100Hz update rate):

$$x_h(k+1) = A_h x_h(k) + B_h(u(k) + \xi_u(k)) \tag{A3}$$

The discrete state space matrices are:

$$A_h = e^{A_{h,cnt}\Delta}; \quad B_h = (\int_0^{\delta} e^{A_{h,cnt}\tau} d\tau)B_{h,cnt} \tag{A4}$$

A.2 Cursor model During brain control the cursor position is determined by integrating the velocity predicted by the BMI filter, v_{BMI} (Eq. 12), after removing potential slow drifts with a first order Butterworth high-pass filter with cutoff frequency of 0.2 Hz [5]. The velocity is predicted from binned spike counts, with bin width of $T = 0.1sec$, so the cursor position p_c is determined from the following difference equation (where the index j denotes the j-th samples at 10Hz update rate):

$$p_c(j+1) = ap_c(j) + bTv_{BMI}(j) \tag{A5}$$

where $a = 0.88, b = 0.94$ are the coefficients of the high-pass filter. The cursor velocity v_c can be computed by taking difference of the cursor position:

$$v_c(j+1) = \frac{p_c(j+1) - p_c(j)}{T} = -\frac{(1-a)}{T}p_c(j) + bv_{BMI}(j) \tag{A6}$$

Denoting by $x_c = [p_c \quad v_c]$ the cursor state (in a single dimension), its discrete dynamics can be expressed as:

$$x_c(j+1) = A_c x_c(j) + B_c v_{BMI}(j) \tag{A7}$$

where

$$A_c = \begin{bmatrix} a & 0 \\ -\frac{(1-a)}{T} & 0 \end{bmatrix}; B_c = \begin{bmatrix} bT \\ b \end{bmatrix}; \tag{A8}$$

During pole control, the same method is used based on the actual rather than the predicted hand velocity, so v_{BMI} in Eq. A5-A7 is replaced with v_h. During brain control, the predicted velocity v_{BMI} is expected to be a distorted reconstruction of hand velocity, i.e., $v_{BMI} = v_h + \xi_{rec}$, where ξ_{rec} is the BMI reconstruction error. In order to evaluate the effect of this reconstruction error explicitly, we simulate noisy pole control, where the v_{BMI} in Eq. A5-A7 is replaced with $v_h + \xi_{rec}$ and the effect of increasing the variance of ξ_{rec} is evaluated.

A.3 Combined model During the BMI experiments, the cursor position was updated at the low rate of 10Hz (as in Eq. A6). The simulation is updated at 100Hz to capture well the continuous dynamics of the hand and its proprioceptive measurements (as in Eq. A3). To support the high update rate of the combined model, the cursor state space is expanded to include 10 samples at 10msec. The resulting 25-dimensional combined state of the hand, cursor and target, r, in each dimension is:

$$x(k) = [x_h'(k) \quad x_c'(k) \quad x_c'(k+1) \quad \ldots \quad x_c'(k+9) \quad r(k)]' \tag{A9}$$

The discrete dynamic equation of the combined system, at 100Hz, is:

$$x(k+1) = Ax(k) + B_u u(k) + B_{BMI} v_{BMI}(k) + \xi_p(k) \tag{A10}$$

where ξ_p is the process noise which is derived from Eq. A3, i.e., $\xi_p = B_h \xi_u$

The matrix $A_{25\times25}$ has a similar structure in both pole and brain control, except for the $A_{2\times4}^{mode}$ (mode is PC or BC, for pole and brain control, respectively) sub-matrix that relates the update in the cursor to the velocity of the hand:

$$A_{25\times25} = \begin{bmatrix} A_{h(4\times4)} & 0_{4\times20} & 0_{4\times1} & \\ 0_{18\times4} & 0_{18\times2} & I_{18\times18} & 0_{18\times1} \\ A_{2\times4}^{mode} & A_{c(2\times2)} & 0_{18\times2} & 0_{2\times1} \\ 0_{1\times4} & 0_{1\times20} & 1 & \end{bmatrix} \tag{A11}$$

where

$$A_{2\times4}^{PC} = [0_{2\times1} \quad B_{c(2\times1)} \quad 0_{2\times2}]; \quad A_{2\times4}^{BC} = [0_{2\times4}] \tag{A12}$$

The vector $B_{u(25\times1)}$ is the same in both pole and brain control, while the vectors $B_{BMI(25\times1)}$ differ

$$B_u = \begin{bmatrix} B_{h(4\times1)} \\ 0_{21\times1} \end{bmatrix}; \quad B_{BMI}^{PC} = [0_{25\times1}]; \quad B_{BMI}^{BC} = \begin{bmatrix} 0_{22\times1} \\ B_{c(2\times1)} \\ 0_{1\times1} \end{bmatrix}; \tag{A13}$$

During normal simulations of pole control the process noise accounts only for the noise in the control signal. The same also holds for brain control, since the other input, v_{BMI}, is generated directly by the

simulated BMI filter, and while it is expected to deviate from the actual velocity, no additional noise is involved. The process noise is assumed to be white zero-mean Gaussian noise, so its covariance matrix can be expressed as:

$$E[\xi_p(j)\xi_p(i)^T] = \Omega^p\delta_{ij}; \quad \Omega^p = \alpha_u^2 B_u B_u' \tag{A14}$$

The combined state defined in Eq. A9 includes 10 samples of the cursor state, which serve as a buffer that keeps each value for 10 steps at the high 100Hz update rate of the combined model. The update rate of the visual measurement of the cursor state is reduced to 10Hz by following the same cursor state as it moves from right to left in this buffer. Specifically, in each dimension, the measurement $y = [p_h \quad v_h \quad p_c \quad v_c]^T$ is extracted from the combined state by a time-varying matrix H_k:

$$y(k) = H_k x(k) + \omega(k) \tag{A15}$$

where

$$H_{k(4\times25)} = \begin{bmatrix} I_{2\times4} & 0_{2\times9} & 0_{2\times2} & 0_{2\times9} & 0_{2\times1} \\ 0_{2\times4} & 0_{2\times2(9-rem(k,10)} & I_{2\times2} & 0_{2\times2(rem(k,10)} & 0_{2\times1} \end{bmatrix}$$

The measurement noise $\omega(k)$ is 4th dimensional (proprioceptive and visual) noise vector, which is assumed to be zero mean white Gaussian noise characterized by the covariance matrix:

$$E[\omega_i\omega_j^T] = \Omega^m\delta_{ij}; \quad \Omega^m = diag([\sigma_{P_p} \quad \sigma_{P_v} \quad \sigma_{V_p} \quad \sigma_{V_v}]) \tag{A16}$$

where $\sigma^2_{P_p}$,$\sigma^2_{P_v}$, are the variance of the proprioceptive (P) measurement noise of the hand position and velocity, respectively, and $\sigma^2_{V_p}$, $\sigma^2_{V_v}$ are the variance of the visual (V) measurement noise of the cursor position and velocity, respectively. The values of these parameters during pole control are specified in Table 1. Same values were used during BCWHM, while the lack of proprioceptive measurements during BCWOHM was modeled by increasing $\sigma^2_{P_p}$,$\sigma^2_{P_v}$ to infinity.

B OPTIMAL STATE ESTIMATION AND FEEDBACK CONTROL

B.1 Optimal state estimation State estimation is based on an internal forward model. The monkey is assumed to be well-adapted to reaching movements in pole control, so the internal forward model is assumed to be identical to the actual hand and cursor model in pole control (Eq. A10 with the matrices for pole control). Under the assumption that the process and measurement noise are Gaussian white noise, optimal state estimation involves the well-known Kalman filter [46], which depends on the presumed covariance matrices of the process and measurement noise. Since the Monkey is assumed to be well adapted to operating in pole control, it is assumed that the Kalman filter is based on the actual covariance matrices of the noise. Thus, the dynamics of the prior and posterior estimated states $\hat{x}_{k|k-1}$ and $\hat{x}_{k|k}$, respectively, the Kalman gains K_k, and the evolution of the covariance matrices of the prior and posterior estimation error $P_{k|k-1}$ and $P_{k|k-1}$, respectively, are given by [46]

$$\hat{x}_{k|k-1} = A\hat{x}_{k-1|k-1} + B_u u_{k-1} \tag{B1}$$
$$P_{k|k-1} \equiv cov[x_k - \hat{x}_{k|k-1}] = AP_{k-1|k-1}A^T + \Omega^p$$
$$K_k = P_{k|k-1}H_k^T(H_k P_{k|k-1} H_k^T + \Omega^m)^{-1}$$
$$\hat{x}_{k|k} = \hat{x}_{k|k-1} + K_k(y_k - H_k\hat{x}_{k|k-1})$$
$$P_{k|k} \equiv cov[x_k - \hat{x}_{k|k}] = (I - K_k H_k)P_{k|k-1}$$

Where A, B_u, and H_k , are the matrices defining the system dynamics and measurements in pole control (given by Eq. A11-A13 and Eq. A15), and Ω^p and Ω^m the actual covariance matrices of the process and measurement noise during pole control, as specified by Eq. A14 and A16, respectively.

B.2 Optimal feedback control (OFC) The model described in Eq. A10 is stabilizable (only the target position is uncontrollable). The optimal control gains are derived from a relevant cost function that accounts for both the goal (i.e. reaching the target, r , and receiving the reward) and the effort. A common cost function has the form:

$$J = \sum_{k=1}^{\infty} \left((x_k - r)^T Q_k (x_k - r) - u_k^T R_k u_k \right) \tag{B2}$$

The matrices Q_k and R_k specify the relative importance of the distance to the target and control effort at the k-th time step, respectively. The latter reflects the cost of high energy consumption and potential large noise associated with large control effort.

The hand movement involves two sub-tasks: (I) reaching the target r by time t_f , and (II) holding the cursor at the target. The cost function for the first sub-task penalizes for distance from the target only at t_f [49]:

$$J_I = (p_c(t_f) - r)^2 + (w_v v_c(t_f))^2 + (w_f f(t_f))^2 + R\sum_{k=1}^{t_f} u_k^T u_k \tag{B3}$$

Where w_v, and w_f are the relative weights for the final velocity and force, compared to the weight for the final position that is one, and R the relative weight of the integrated control effort. The target, r , is included in the state vector specified in Eq. A9 so defining $p_Q^T = [0_{1\times4} \quad 1 \quad 0_{1\times19} \quad (-1)]$, the first term in Eq. B2 can be expressed as $x_{t_f}^T(p_Q p_Q^T)x_{t_f}$. Thus, Eq. B3 can be expressed as Eq. B2 with $R_k = R$, $Q_{1\cdots t_f-1} = 0$ and $Q_{t_f} = p_Q p_Q^T + v_Q v_Q^T + f_Q f_Q^T$ where $v_Q^T = [0_{1\times5} \quad w_v \quad 0_{1\times19}]$ and $f_Q^T = [0_{1\times2} \quad w_f \quad 0_{1\times22}]$.

The cost function for the second sub-task penalizes for distance from the target for all future times, and is given by

$$J_{II} = \sum_{k=t_f+1}^{\infty} \left((p_c(k) - r)^2 + (w_v v_c(k))^2 + (w_f f(k))^2 \right) + R \sum_{k=t_f+1}^{\infty} u_k^T u_k \tag{B4}$$

This can be expressed as Eq. B1, with $R_k = R$ and $Q_{t_f+1\cdots\infty} = p_Q p_Q^T + v_Q v_Q^T + f_Q f_Q^T$

The OFC [46]:

$$u_k = -L_k \hat{x}_{k|k} \tag{B5}$$

is computed from the posterior estimated state $\hat{x}_{k|k}$, Eq. B1, and the feedback gain matrix L_k (in the single dimension case, considered here, this is a raw vector). Under the assumption that the noise is Gaussian and independent of the control signal, the feedback gains are given by Linear Quadratic Gaussian (LQG) controller

$$L_k = (R + B_u^T S_{k+1} B_u)^{-1} B_u^T S_{k+1} A \tag{B6}$$

Where S_k is determined by the following matrix Riccati difference equation that runs backward in time:

$$S_i = A^T (S_{i+1} - S_{i+1} B_u (B_u^T S_{i+1} B_u + R)^{-1} B_u^T S_{i+1}) A + Q_i \tag{B7}$$

For the infinite horizon problem defined in Eq. B2, B7 reduces to the discrete algebraic Riccati equation:

$$S_\infty = A^T (S_\infty - S_\infty B_u (B_u^T S_\infty B_u + R)^{-1} B_u^T S_\infty) A + Q_\infty \tag{B8}$$

which can be solved using MATLAB DARE solver.

C POM ANALYSIS : PROOF OF PROPOSITION

Assuming the cumulative spike-rate, Γ , encodes a linear combination of the estimated state, it can be expressed as the output of an extended linear system whose state $\tilde{x}_k = [x_k \quad \hat{x}_{k|k} \quad 1]'$ includes the dynamics of both the actual and estimated states (of the hand and cursor). In order to derive the dynamics of the extended system, we first note that the first and fourth lines in Eq. B1 can be combined to express the dynamics of the estimated state as:

$$\hat{x}_{k|k} = (I - K_k H_k) A \hat{x}_{k-1|k-1} + (I - K_k H_k) B_u u_{k-1} + K_k y_k \tag{C1}$$

Inserting Eq. B5 (control law) and Eq. A15 (measurement equation) into C1, results in the dynamic equation for the combined state: :

$$\tilde{x}_{k+1} = \begin{bmatrix} A & -BL_k & 0 \\ K_k H_k A & (I - K_{k+1} H_k)(A - BL_k) - K_{k+1} H_k B L_k & 0 \\ 0 & 0 & 1 \end{bmatrix} \tilde{x}_k + \begin{bmatrix} \xi_k \\ K_{k+1} H_k \xi_k + K_{k+1} \omega_{k+1} \\ 0 \end{bmatrix} \tag{C2}$$

Under the restrictions of TA1, the speed and the magnitude of the control are excluded from Eq.11. Hence, and given that the control signal is proportional to the estimated state (Eq. B5), the cumulative spike-rate can be expressed as the output of the combined system:

$$\Gamma = ([0_{1\times25} \quad -\beta_u L_k \quad \beta_0] + [0_{1\times29} \quad \beta_{\hat{p}} \quad \beta_{\hat{v}} \quad 0_{1\times20}]) \begin{bmatrix} x_k \\ \hat{x}_{k|k} \\ 1 \end{bmatrix} \tag{C3}$$

where $\beta_{\hat{p}}$, $\beta_{\hat{v}}$ and β_u are the components of the tuning weight vector β corresponding to single dimension (x or y) of the estimated position, estimated velocity and control. As mentioned above, actual simulations

were conducted in two independent dimensions, so the bin-rate is a linear combination of the extended states in both dimensions. Defining:

$$\tilde{A}_k = \begin{bmatrix} A & -BL_k & 0 & \\ & K_kH_kA & (I-K_{k+1}H_k)(A-BL_k)-K_{k+1}H_kBL_k & 0 \\ & 0 & 0 & 1 \end{bmatrix} \tag{C4}$$

$$\tilde{H}_k = ([0_{1\times 25} \quad -\beta_u L_k \quad \beta_0] + [0_{1\times 29} \quad \beta_{\hat{p}} \quad \beta_{\hat{v}} \quad 0_{1\times 20}]$$

$$\tilde{\xi} = \begin{bmatrix} \xi_k \\ K_{k+1}H_k\xi_k + K_{k+1}\omega_{k+1} \\ 0 \end{bmatrix}$$

The dynamics of the extended system can be expressed as a linear system:

$$\begin{aligned} \tilde{x}_{k+1} &= \tilde{A}_k\tilde{x}_k + \tilde{\xi} \\ \Gamma_k &= \tilde{H}_k\tilde{x}_k \end{aligned} \tag{C5}$$

The process noise of the extended system $\tilde{\xi}$ captures both the process noise of the actual system and the estimation error. Since both are assumed to be white Gaussian noise, so is $\tilde{\xi}$, where

$$E[\tilde{\xi}_i\tilde{\xi}_j^T] = \Omega_i^{\tilde{p}}\delta_{ij}; \qquad \Omega_k^{\tilde{p}} = \begin{bmatrix} \Omega_k^p & 0 & 0 \\ 0 & K_{k+1}H_k\Omega_k^pH_k^TK_{k+1}^T + K_{k+1}\Omega^mK_{k+1}^T & 0 \\ 0 & 0 & 0 \end{bmatrix} \tag{C6}$$

Claim: Let $\tilde{x}_k^L$ and $\tilde{x}_k^H$ denote the extended states of two systems described by C6 with the same parameters except for the process noise of the actual system that is either Ω_L^p or Ω_H^p , respectively, where $\Omega_H^p \succcurlyeq \Omega_L^p$. then $E_m[\tilde{x}_k^H] = E_m[\tilde{x}_k^L]$ but $E_m[\tilde{x}_k^H(\tilde{x}_k^H)^T] \succcurlyeq E_m[\tilde{x}_k^L(\tilde{x}_k^L)^T]$, where $E_m[\cdot]$ denotes ensemble average over different movements starting from the same distribution of initial conditions , and $\cdot \succcurlyeq 0$ denotes positive semidefinite matrix (psd). Note that although the actual process noise is different, the Kalman gains of the two systems are assumed to be the same, as in the case when they are both optimized for the same internal model of the process noise.

Proof: According to a general property of psd matrices, $Z \succcurlyeq 0$ implies that $VZV^T \succcurlyeq 0$,[25], [52]. Hence, given that the Kalman gains K_k, the covariance of the measurement noise Ω^m , and the output matrix H_k are the same in the two systems, $\Omega_H^{\tilde{p}} - \Omega_L^{\tilde{p}} \succcurlyeq 0$ implies that for each k, $K_{k+1}H_k\Omega_H^pH_k^TK_{k+1}^T \succcurlyeq K_{k+1}H_k\Omega_k^LH_k^TK_{k+1}^T$ while $K_k\Omega^mK_k^T$ is the same. Hence, $\Omega_H^p - \Omega_L^p \succcurlyeq 0$ implies that $\Omega_H^{\tilde{p}} - \Omega_L^{\tilde{p}} \succcurlyeq 0$.

We proceed by induction:

Initial step: under the conditions of the claim, show that $E_m[\tilde{x}_1^H] = E_m[\tilde{x}_1^L]$ and $E_m[\tilde{x}_1^H(\tilde{x}_1^H)^T] \succcurlyeq E_m[\tilde{x}_1^L(\tilde{x}_1^L)^T]$.

Proof of initial step: Since the distribution of the initial conditions is the same, $E_m[\tilde{x}_0^H] = E_m[\tilde{x}_0^L] \equiv E_m[\tilde{x}_0]$ and $E_m[\tilde{x}_0^H(\tilde{x}_0^H)^T] = E_m[\tilde{x}_0^L(\tilde{x}_0^L)^T] \equiv E_m[\tilde{x}_0(\tilde{x}_0)^T]$. According to Eq. C5

$E_m[\tilde{x}_1^{H|L}] = \tilde{A}_0 E_m[\tilde{x}_0]$ and hence $E_m[\tilde{x}_1^H] = E_m[\tilde{x}_1^L]$. Eq. C5 also implies that $E_m[\tilde{x}_1^{H|L}(\tilde{x}_1^{H|L})^T] = \tilde{A}_0 E_m[\tilde{x}_0^{H|L}(\tilde{x}_0^{H|L})^T]\tilde{A}_0^T + \Omega^{\tilde{p}}_{H|L}$, and hence $E_m[\tilde{x}_1^H(\tilde{x}_1^H)^T] - E_m[\tilde{x}_1^L(\tilde{x}_1^L)^T] = \Omega^{\tilde{p}}_H - \Omega^{\tilde{p}}_L \succcurlyeq 0$.

Induction step: assuming by induction that $E_m[\tilde{x}_k^H] = E_m[\tilde{x}_k^L]$ and $E_m[\tilde{x}_k^H(\tilde{x}_k^H)^T] \succcurlyeq E_m[\tilde{x}_k^L(\tilde{x}_k^L)^T]$ show that $E_m[\tilde{x}_{k+1}^H] = E_m[\tilde{x}_{k+1}^L]$ and $E_m[\tilde{x}_{k+1}^H(\tilde{x}_{k+1}^H)^T] \succcurlyeq E_m[\tilde{x}_{k+1}^L(\tilde{x}_{k+1}^L)^T]$.

Proof of induction step: According to Eq. C5, $E_m[\tilde{x}_{k+1}^{H|L}] = \tilde{A}_k E_m[\tilde{x}_k^{H|L}]$ so the first induction assumption implies that $E_m[\tilde{x}_{k+1}^H] = E_m[\tilde{x}_{k+1}^L]$. Eq. C5 also implies that $E_m[\tilde{x}_{k+1}^{H|L}(\tilde{x}_{k+1}^{H|L})^T] = \tilde{A}_k E_m[\tilde{x}_k^{H|L}(\tilde{x}_k^{H|L})^T]\tilde{A}_k^T + \Omega^{\tilde{p}}_{H|L}$ so $E_m[\tilde{x}_{k+1}^H(\tilde{x}_{k+1}^H)^T] - E_m[\tilde{x}_{k+1}^L(\tilde{x}_{k+1}^L)^T] = \tilde{A}_k(E_m[\tilde{x}_k^H(\tilde{x}_k^H)^T] - E_m[\tilde{x}_k^L(\tilde{x}_k^L)^T])\tilde{A}_k^T + (\Omega^{\tilde{p}}_H - \Omega^{\tilde{p}}_L)$. The second induction assumption (and the above general property of psd matrices) implies that the first term is also psd, and as was shown above, that the second term is psd. Hence, $E_m[\tilde{x}_{k+1}^H(\tilde{x}_{k+1}^H)^T] \succcurlyeq E_m[\tilde{x}_{k+1}^L(\tilde{x}_{k+1}^L)^T]$.

End of claim.

Proposition C1: Let Γ_k^L and Γ_k^H be the cumulative spike-rates generated by two systems described by Eq. C6 with the same parameters except for the process noise that is either Ω^p_L or Ω^p_H, respectively, where $\Omega^p_H \succcurlyeq \Omega^p_L$, then $E_m[\Gamma_k^H] = E_m[\Gamma_k^L]$ but $E_m[(\Gamma_k^H)^2] \geq E_m[(\Gamma_k^L)^2]$, where $E_m[\cdot]$ denotes ensemble average over different movements starting from the same distribution of initial conditions.

Proof: The two parts of Proposition C1 follow from the two parts of the claim, by noting that Eq. C5 implies that $E_m[\Gamma_k^{H|L}] = \tilde{H} E_m[\tilde{x}_k^{H|L}]$ and $E_m[(\Gamma_k^{H|L})^2] = \tilde{H} E_m[\tilde{x}_k^{H|L}(\tilde{x}_k^{H|L})^T]\tilde{H}^T$. By the first part of the claim $E_m[\tilde{x}_k^H] = E_m[\tilde{x}_k^L]$, and hence $E_m[\Gamma_k^H] = E_m[\Gamma_k^L]$. Recalling that $Z \succcurlyeq 0$ implies that $VZV^T \succcurlyeq 0$, [25], [52], the second part of the claim, i.e., that $E_m[\tilde{x}_k^H(\tilde{x}_k^H)^T] \succcurlyeq E_m[\tilde{x}_k^L(\tilde{x}_k^L)^T]$ implies that $E_m[(\Gamma_k^H)^2] \geq E_m[(\Gamma_k^L)^2]$.

End of proposition C1.

Proposition C2: Let POM^L and POM^H be the POM (Eq. 1) of the spike-counts generated by DSPP from cumulative spike-rates Γ_k^L and Γ_k^H , respectively. If $E_m[(\Gamma_k^H)] = E_m[(\Gamma_k^L)]$ and $E_m[(\Gamma_k^H)^2] \geq E_m[(\Gamma_k^L)^2]$ for each k, where $Em[\cdot]$ denotes ensemble mean over different movements starting from the same distribution of initial conditions, then $POM^H \geq POM^L$.

Proof: For DSPP, Eq. 2 implies that the POM, defined in Eq. 1, can be expressed as a function of the statistics of the cumulative spike-rate Γ as:

$$POM = \frac{var[\Gamma]}{var[\Gamma] + E[\Gamma]} = \frac{E[\Gamma^2] - (E[\Gamma])^2}{E[\Gamma^2] - (E[\Gamma])^2 + E[\Gamma]} \tag{C7}$$

where the ensemble averages are over different movements and time-steps. In particular,

$$E[\Gamma^2] = \lim_{T\to\infty}\left(\frac{1}{T}\sum_{i=1}^{T} E_m[\Gamma_i^2]\right) \tag{C8}$$

Hence, the condition that $E_m[\Gamma_k^H] = E_m[\Gamma_k^L]$ for each k implies that $E[\Gamma^H] = E[\Gamma^L]$, while the condition that $E_m[(\Gamma_k^H)^2] \geq E_m[(\Gamma_k^L)^2]$ for each k, implies that $E[(\Gamma^H)^2] \geq E[(\Gamma^L)^2]$. Given that $E[\Gamma^H] = E[\Gamma^L]$ is a positive constant, Eq. C7 implies that the POM increases with $E[\Gamma^2]$. Thus, $E[(\Gamma^H)^2] \geq E[(\Gamma^L)^2]$ implies that $POM^H \geq POM^L$.

End of proposition C2.

REFERENCES

[1] Ashe, J. (1997), Force and the motor cortex, *Behavioural brain research*, 87, 2, 255–269

[2] Ashe, J. and Georgopoulos, A. P. (1994), Movement parameters and neural activity in motor cortex and area 5, *Cerebral Cortex*, 4, 6, 590–600

[3] Bensmaia, S. J. and Miller, L. E. (2014), Restoring sensorimotor function through intracortical interfaces: progress and looming challenges, *Nature reviews Neuroscience*, 15, 5, 313–325

[4] Brown, E. N., Kass, R. E., and Mitra, P. P. (2004), Multiple neural spike train data analysis: state-of-the-art and future challenges, *Nature neuroscience*, 7, 5, 456–461

[5] Carmena, J. M., Lebedev, M. a., Crist, R. E., O'Doherty, J. E., Santucci, D. M., Dimitrov, D. F., et al. (2003), Learning to control a brain-machine interface for reaching and grasping by primates., *PLoS biology*, 1, 2, E42, doi:10.1371/journal.pbio.0000042

[6] Chang, Y. H., Chen, M., Shanechi, M., Carmena, J. M., and Tomlin, C. (2014), A design of neural decoder by reducing discrepancy between Manual Control (MC) and Brain Control (BC), *2014 European Control Conference (ECC)*, , Mc, 516–521, doi:10.1109/ECC.2014.6862547

[7] Churchland, A. K., Kiani, R., Chaudhuri, R., Wang, X.-J., Pouget, A., and Shadlen, M. N. (2011), Variance as a signature of neural computations during decision making., *Neuron*, 69, 4, 818–31, doi:10.1016/j.neuron.2010.12.037

[8] Crevecoeur, F. and Scott, S. H. (2013), Priors engaged in long-latency responses to mechanical perturbations suggest a rapid update in state estimation, *PLoS computational biology*, 9, 8, e1003177

[9] Cunningham, J. P., Nuyujukian, P., Gilja, V., Chestek, C. A., Ryu, S. I., and Shenoy, K. V. (2011), A closed-loop human simulator for investigating the role of feedback control in brain-machine interfaces, *Journal of Neurophysiology*, 105, 4, 1932–1949

[10] Dayan, P. and Abbott, L. F. (2001), Theoretical neuroscience (Cambridge, MA: MIT Press)

[11] DiGiovanna, J., Mahmoudi, B., Fortes, J., Principe, J. C., and Sanchez, J. C. (2009), Coadaptive brain–machine interface via reinforcement learning, *Biomedical Engineering, IEEE Transactions on*, 56, 1, 54–64

[12] Doeringer, J. A. and Hogan, N. (1998), Intermittency in preplanned elbow movements persists in the absence of visual feedback, *Journal of Neurophysiology*, 80, 4, 1787–1799

[13] Evarts, E. V. (1968), Relation of pyramidal tract activity to force exerted during voluntary movement, *J Neurophysiol*, 31, 1, 14–27

[14] Flash, T. and Hogan, N. (1985), The coordination of arm movements: an experimentally confirmed mathematical model, *The journal of Neuroscience*, 5, 7, 1688–1703

[15] Friston, K., Mattout, J., and Kilner, J. (2011), Action understanding and active inference, *Biological cybernetics*, 104, 1-2, 137–160

[16] Friston, K. J., Daunizeau, J., Kilner, J., and Kiebel, S. J. (2010), Action and behavior: a free-energy formulation, *Biological cybernetics*, 102, 3, 227–260

[17] Gawthrop, P., Loram, I., Lakie, M., and Gollee, H. (2011), Intermittent control: a computational theory of human control., *Biological cybernetics*, 104, 1-2, 31–51, doi:10.1007/s00422-010-0416-4

[18] Geisler, W. and Albrecht, D. (1995), Bayesian analysis of identification performance in monkey visual cortex: nonlinear mechanisms and stimulus certainty, *Vision research*, 35, 19, 2723–2730

[19] Georgopoulos, A., Caminiti, R., and Kalaska, J. (1984), Static spatial effects in motor cortex and area 5: quantitative relations in a two-dimensional space, *Experimental Brain Research*, 54, 3, 446–454

[20] Georgopoulos, A. P., Ashe, J., Smyrnis, N., and Taira, M. (1992), The motor cortex and the coding of force, *Science*, 256, 5064, 1692–1695

[21] Georgopoulos, A. P., Kalaska, J. F., Caminiti, R., and Massey, J. T. (1982), On the relations between the direction of two-dimensional arm movements and cell discharge in primate motor cortex, *The Journal of Neuroscience*, 2, 11, 1527–1537

[22] Georgopoulos, A. P., Schwartz, A. B., and Kettner, R. E. (1986), Neuronal population coding of movement direction, *Science*, 233, 4771, 1416–1419

[23] Harris, C. M. and Wolpert, D. M. (1998), Signal-dependent noise determines motor planning, *Nature*, 394, 6695, 780–784
[24] Hendrix, C. M., Mason, C. R., and Ebner, T. J. (2009), Signaling of grasp dimension and grasp force in dorsal premotor cortex and primary motor cortex neurons during reach to grasp in the monkey., *Journal of neurophysiology*, 102, 1, 132–45, doi:10.1152/jn.00016.2009
[25] Horn, R. A. and Johnson, C. R. (2012), Matrix analysis (Cambridge university press)
[26] Johnson, D. H. (1996), Point process models of single-neuron discharges, *Journal of computational neuroscience*, 3, 4, 275–299
[27] Jordan, M. I. and Rumelhart, D. E. (1992), Forward Models: Supervised Learning with a Distal Teacher, *Cognitive Science*, 16, 3, 307–354, doi:10.1207/s15516709cog1603_1
[28] Kalaska, J. F. (2009), From intention to action: motor cortex and the control of reaching movements, in Progress in Motor Control (Springer), 139–178
[29] Kalaska, J. F., Cohen, D., Hyde, M. L., and Prud'Homme, M. (1989), A comparison of movement direction-related versus load direction-related activity in primate motor cortex, using a two-dimensional reaching task, *The Journal of neuroscience*, 9, 6, 2080–2102
[30] Kawato, M., Furukawa, K., and Suzuki, R. (1987), A hierarchical neural-network model for control and learning of voluntary movement, *Biological cybernetics*, 57, 3, 169–185
[31] Kuo, A. D. (1995), An optimal control model for analyzing human postural balance, *Biomedical Engineering, IEEE Transactions on*, 42, 1, 87–101
[32] Lebedev, M. A., Carmena, J. M., O'Doherty, J. E., Zacksenhouse, M., Henriquez, C. S., Principe, J. C., et al. (2005), Cortical ensemble adaptation to represent velocity of an artificial actuator controlled by a brain-machine interface, *The Journal of neuroscience*, 25, 19, 4681–4693
[33] Mahmoudi, B., Pohlmeyer, E. A., Prins, N. W., Geng, S., and Sanchez, J. C. (2013), Towards autonomous neuroprosthetic control using hebbian reinforcement learning, *Journal of neural engineering*, 10, 6, 066005
[34] Messier, J. and Kalaska, J. F. (2000), Covariation of primate dorsal premotor cell activity with direction and amplitude during a memorized-delay reaching task, *Journal of Neurophysiology*, 84, 1, 152–165
[35] Miall, R. and Wolpert, D. M. (1996), Forward models for physiological motor control, *Neural networks*, 9, 8, 1265–1279
[36] Moran, D. W. and Schwartz, A. B. (1999), Motor cortical representation of speed and direction during reaching, *Journal of Neurophysiology*, 82, 5, 2676–2692
[37] Nicolelis, M. A. (2001), Actions from thoughts, *Nature*, 409, 6818, 403–407
[38] Paninski, L., Fellows, M. R., Hatsopoulos, N. G., and Donoghue, J. P. (2004), Spatiotemporal tuning of motor cortical neurons for hand position and velocity., *Journal of neurophysiology*, 91, 1, 515–32, doi:10.1152/jn.00587.2002
[39] Schwartz, A. B. (2004), Cortical neural prosthetics., *Annual review of neuroscience*, 27, 487–507, doi:10.1146/annurev.neuro.27.070203.144233
[40] Shadmehr, R. and Krakauer, J. W. (2008), A computational neuroanatomy for motor control., *Experimental brain research*, 185, 3, 359–81, doi:10.1007/s00221-008-1280-5
[41] Shanechi, M. M., Williams, Z. M., Wornell, G. W., Hu, R. C., Powers, M., and Brown, E. N. (2013), A real-time brain-machine interface combining motor target and trajectory intent using an optimal feedback control design, *PloS one*, 8, 4, e59049
[42] Shanechi, M. M., Wornell, G. W., Williams, Z. M., and Brown, E. N. (2013), Feedback-controlled parallel point process filter for estimation of goal-directed movements from neural signals, *Neural Systems and Rehabilitation Engineering, IEEE Transactions on*, 21, 1, 129–140
[43] Shenoy, K. V., Sahani, M., and Churchland, M. M. (2013), Cortical control of arm movements: a dynamical systems perspective, *Annual review of neuroscience*, 36, 337–359
[44] Simon, H. A. (1956), Rational choice and the structure of the environment., *Psychological review*, 63, 2, 129
[45] Snyder, D. L. (1975), Random point processes (Wiley)
[46] Stengel, R. F. (2012), Optimal control and estimation (Courier Dover Publications)

[47] Taylor, D. M., Tillery, S. I. H., and Schwartz, A. B. (2002), Direct cortical control of 3d neuroprosthetic devices, *Science*, 296, 5574, 1829–1832
[48] Todorov, E. (2000), Direct cortical control of muscle activation in voluntary arm movements: a model, *Nature neuroscience*, 3, 4, 391–398
[49] Todorov, E. (2005), Stochastic optimal control and estimation methods adapted to the noise characteristics of the sensorimotor system., *Neural computation*, 17, 5, 1084–108, doi:10.1162/0899766053491887
[50] Todorov, E. and Jordan, M. I. (2002), Optimal feedback control as a theory of motor coordination., *Nature neuroscience*, 5, 11, 1226–35, doi:10.1038/nn963
[51] Tolhurst, D. J., Movshon, J., and Dean, A. (1983), The statistical reliability of signals in single neurons in cat and monkey visual cortex, *Vision research*, 23, 8, 775–785
[52] Van den Bos, A. (2007), Parameter estimation for scientists and engineers (John Wiley & Sons)
[53] Winter, D. A. (1991), Biomechanics and motor control of human gait: normal, elderly and pathological
[54] Wolpert, D., Ghahramani, Z., and Jordan, M. (1995), Are arm trajectories planned in kinematic or dynamic coordinates? An adaptation study, *Experimental Brain Research*, 103, 3, 460–470, doi:10.1007/BF00241505
[55] Wolpert, D. M. and Ghahramani, Z. (2000), Computational principles of movement neuroscience, *nature neuroscience*, 3, 1212–1217
[56] Wu, W., Gao, Y., Bienenstock, E., Donoghue, J. P., and Black, M. J. (2006), Bayesian population decoding of motor cortical activity using a Kalman filter., *Neural computation*, 18, 1, 80–118, doi:10.1162/089976606774841585
[57] Zacksenhouse, M., Lebedev, M. a., Carmena, J. M., O'Doherty, J. E., Henriquez, C., and Nicolelis, M. a. L. (2007), Cortical modulations increase in early sessions with brain-machine interface., *PloS one*, 2, 7, e619, doi:10.1371/journal.pone.0000619
[58] Zacksenhouse, M., Lebedev, M. a., and Nicolelis, M. a. L. (2014), Signal-independent timescale analysis (SITA) and its application for neural coding during reaching and walking., *Frontiers in computational neuroscience*, 8, August, 91, doi:10.3389/fncom.2014.00091
[59] Zacksenhouse, M., Nemets, S., and Nicolelis, M. (2008), Strategies for neural ensemble data analysis for brain–machine interface (bmi) applications, *Methods for Neural Ensemble Recordings*, 57–82
